# Acid‐Degradable Hydrogen‐Generating Metal‐Organic Framework for Overcoming Cancer Resistance/Metastasis and Off‐Target Side Effects

**DOI:** 10.1002/advs.202101965

**Published:** 2022-01-31

**Authors:** Xianxian Yao, Danyang Chen, Bin Zhao, Binru Yang, Zhaokui Jin, Mingjian Fan, Geru Tao, Shucun Qin, Wuli Yang, Qianjun He

**Affiliations:** ^1^ State Key Laboratory of Molecular Engineering of Polymers and Department of Macromolecular Science Fudan University Shanghai 200433 China; ^2^ School of Biomedical Engineering Health Science Center Shenzhen University Shenzhen Guangdong 518060 China; ^3^ Center of Hydrogen Science Shanghai Jiao Tong University Shanghai 200240 China; ^4^ Institute of Atherosclerosis Taishan Institute for Hydrogen Biological Medicine Shandong First Medical University and Shandong Academy of Medical Sciences Taian Shandong 271000 China

**Keywords:** controlled release, drug delivery, hydrogen therapy, metal‐organic framework, metastasis, multidrug resistance, nanomedicine

## Abstract

The development of stimuli‐responsively degradable porous carriers for both controlled drug release and high biosafety is vitally important to their clinical translation, but still challenging at present. A new type of porphyrin–iron metal organic framework (Fe‐MOF) nanocrystals is engineered here as acid‐degradable drug carrier and hydrogen donor by the coordination between porphyrin and zero‐valence Fe atom. Fe‐MOF nanocrystals exhibit excellent acid‐responsive degradation for H_2_ generation and simultaneous release of the loaded drug for combined hydrogen‐chemotherapy of cancer multidrug resistance (MDR) and metastasis and for local hydrogen eradication of the off‐target induced toxic side effects of the drug to normal cells/tissues. Mechanistically, released H_2_ assists chemotherapeutic drug to efficiently inhibit cancer metastasis by immunoactivating intratumoral M1‐phenotype macrophages and consequently downregulating the expression of metastasis‐related matrix metalloproteinase‐2 (MMP‐2) and can also downregulate the expressions of both P‐glycoprotein (P‐gp) protein and adenosine triphosphate (ATP) in MDR cancer cells to sensitize chemotherapeutic drug for enhanced damage to mitochondria and DNA. High anti‐MDR/antimetastasis efficacies and high biocompatibility endow Fe‐MOF nanocrystals and the Fe‐MOF‐based nanomedicine with high potential for clinical translation.

## Graphical abstract

Based on doxorubicin hydrochloride (DOX)‐encapsulated porphyrin‒iron metal organic framework (DOX@Fe‐MOF), hydrogen gas can play a double‐side (Yin−Yang) role as it is proinflammatory to stand with toxic DOX against cancer cells, but is also anti‐inflammatory to counteract toxic DOX for normal cells, which ingeniously addresses the unavoidable nanomedicine off‐target induced toxic side effect of chemotherapeutic agents to normal cells/tissues.

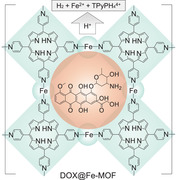

## 1 Introduction

By virtue of drug carriers, the construction of drug delivery systems brings obvious benefits for therapy efficacy enhancement and side effect attenuation by targeted drug delivery and controlled drug release.^[^ ^1^, ^2^, ^3^ ^]^ Organic carriers are characteristic with high biocompatibility and biodegradability, but their physicochemical stability is relatively poor and their functionality is usually single.^[^ ^4^, ^5^, ^6^ ^]^ Comparatively, inorganic carriers have relatively higher physicochemical stability and multifunctionality, but their biodegradation is markedly slower.^[^ ^7^, ^8^ ^]^ Fortunately, metal organic framework (MOF), which is composed of inorganic metal subunit and organic ligand, has emerged as a new type of excellent drug carrier combining the advantages of traditional organic and inorganic carriers and overcoming their individual disadvantages.^[^ ^9^, ^10^, ^11^, ^12^ ^]^ MOF provides a platform to integrate a variety of optical/pH/magnetic/thermal properties by introducing multifunctional metals/ligands, and high porosity favors high drug payload, in great support of controlled drug release.^[^ ^13^, ^14^, ^15^, ^16^, ^17^, ^18^ ^]^ However, it is vitally important but still challenging to obtain the balance between high stability and good biodegradation. Responsive degradation of MOF for controlled drug release at the site of focus is one of promising solutions.

Hydrogen gas (H_2_), a clean energy resource, has received much attention in the forefront of the biomedical field in the treatment of many diseases including cancer, inflammatory, and cardiovascular diseases.^[^ ^19^, ^20^, ^21^, ^22^, ^23^ ^]^ Hydrogen molecules can induce apoptosis of cancer cells, but also assist other therapeutics for enhancing the anticancer outcome by codelivering hydrogen and other therapeutic agents.^[^ ^24^, ^25^, ^26^, ^27^ ^]^ Carboxymethyl cellulose‐coated Fe (Fe@CMC) nanoparticles as hydrogen donors were designed for the tumor‐targeted acid‐responsive release of H_2_, but it is difficult to load amounts of drug on Fe@CMC.^[^ ^28^ ^]^ Moreover, we constructed a nanoscale porphyrin‒palladium MOF for hydrogen delivery, but its hydrogen‐loading capacity was limited. In order to achieve efficient codelivery of hydrogen and drugs, this work proposed to construct a new type of MOF based on Fe and porphyrin for the acid‐responsive decomposition of Fe(0) into hydrogen gas and for the corelease of loaded drug molecules to realize high efficacy of combined therapy.

**Scheme 1:**
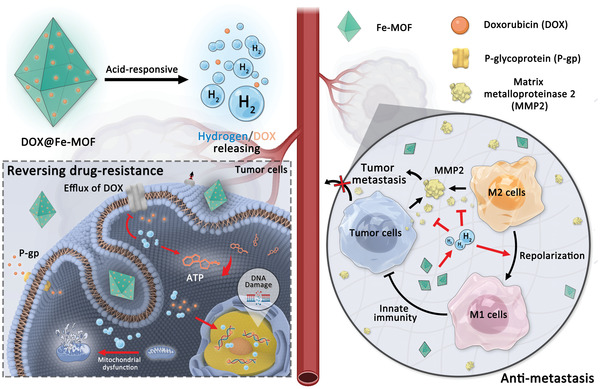
Schematic illustration of acid‐responsive hydrogen/drug release from DOX@Fe‐MOF nanocrystals for overcoming cancer resistance/metastasis.

In this work, we designed and synthesized a new type of porphyrin‒Fe‐MOF with defined octahedral morphology and nanoscale size by the coordination between 5,10,15,20‐tetrakis(4‐pyridyl)‐21H,23H‐porphine (TPyP) and zero‐valence Fe atom for the first time (**Scheme** **1**). Three aspects of advances of this work are worth to be highlighted. 1) *Platform Advance*: The developed Fe‐MOF nanocrystals are an excellent drug/hydrogen delivery platform to play a dual role as both acid‐degradable drug carrier and hydrogen donor, enabling acid‐controlled hydrogen/drug corelease (doxorubicin hydrochloride (DOX)@Fe‐MOF) for the facile combination of hydrogen‐chemotherapy. 2) *Performance Advance*: Fe‐MOF nanocrystals exhibited high biocompatibility as both porphyrin and Fe have been approved for clinical applications by the U.S. Food and Drug Administration (FDA)^[^ ^29^, ^30^, ^31^ ^]^ and the constructed DOX@Fe‐MOF nanomedicine possesses high biosafety by virtue of the chemotherapeutic toxic side effect‐attenuated capability of hydrogen gas, holding a potential for clinical translation. Intriguingly, we have discovered that hydrogen gas can play a double‐side (Yin−Yang) role as it is proinflammatory to stand with toxic DOX against cancer cells, but is also anti‐inflammatory to counteract toxic DOX for normal cells, which ingeniously addressed the unavoidable nanomedicine off‐target induced toxic side effect of chemotherapeutic agents to normal cells/tissues. Moreover, hydrogen gas released from Fe‐MOF efficiently sensitized chemotherapy to achieve high tumor‐inhibition and anti‐multidrug resistance (MDR) efficacies and also assisted chemotherapy to efficiently enhance the efficacy of antimetastasis. 3) *Mechanistic Advance*: we have discovered new mechanisms for combined hydrogen‐chemotherapy of MDR and metastasis. As to antimetastasis, we have here discovered that hydrogen gas can immunoactivate intratumoral M1‐phenotype macrophages to downregulate the expression of matrix metalloproteinase‐2 (MMP‐2) for assisting chemotherapy to inhibit metastasis for the first time. As to anti‐MDR, we have here discovered that hydrogen gas can downregulate the over‐expression of P‐gp by inhibiting the energy of cancer cells to sensitize MDR cancer cells for the first time. In addition, our recognition of the double‐side (proinflammatory in tumor and anti‐inflammatory in normal tissues) role of hydrogen gas is greatly valuable for utilization of hydrogen gas to assist disease treatment.

## 2 Results and Discussion

### 2.1 Synthesis and Characterization of Fe‐MOF Nanocrystals

**Figure 1:**
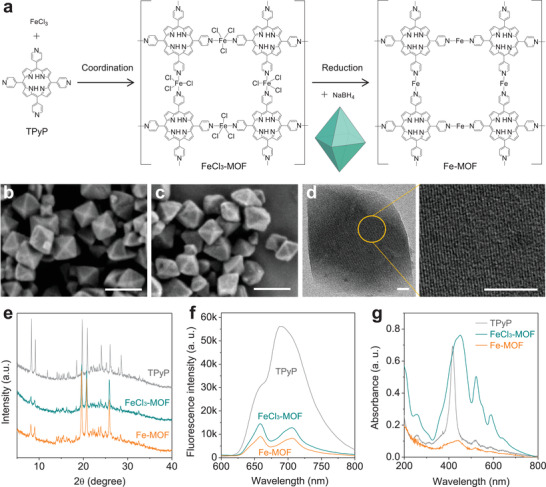
Synthesis route and morphology, structural and optical characterization of Fe‐MOF nanocrystals. A) Schematic illustration of the synthesis route of Fe‐MOF nanocrystals, SEM images of b) FeCl_3_‐MOF and c) Fe‐MOF, high‐resolution TEM images of d) Fe‐MOF, e) XRD spectra, f) UV–vis absorption spectra, and g) fluorescence emission spectra of free TPyP, FeCl_3_‐MOF, and Fe‐MOF at the same molar concentration. Excitation wavelength: 452 nm. Scale bars in (c) and (d) correspond to 200 and 20 nm, respectively.

FeCl_3_‐MOF nanocrystals were prepared with FeCl_3_ and TPyP by a coordination method and further reduced by sodium borohydride into Fe‐MOF nanocrystals (**Figure** **1**a). After reduction, chlorine atoms were eliminated, producing homogeneously dispersed zero‐valence Fe atoms. Through scanning electron microscopy (SEM) observation, both FeCl_3_‐MOF and Fe‐MOF nanocrystals exhibited a kind of octahedron‐like morphology (Figure 1b,c) as well as uniform size of about 120 nm in diameter in favor of cellular uptake. From dynamic light scattering (DLS) data (Figure S1, Supporting Information), both FeCl_3_‐MOF and Fe‐MOF nanocrystals exhibited good dispersion in water and a uniform hydrated diameter of about 180 nm, favoring passive tumor‐targeted delivery. As observed in the high‐resolution transmission electron microscopy (TEM) image (Figure 1d), the lattice fringes of the Fe‐MOF nanocrystal were arranged regularly, indicating a single crystal structure. In addition, powder X‐ray diffraction (XRD) and small‐angle X‐ray scattering (SAXS) were also performed to investigate the change in crystal structure before and after coordination (Figure 1e and Figure S2, Supporting Information). From Figure 1e and Table S1 in the Supporting Information, it can be found that FeCl_3_‐MOF nanocrystals maintained the crystal structure of TPyP with an orthorhombic crystal system but exhibited a slightly expanded crystal lattice, owing to the coordination/incorporation of iron (Table S1, Supporting Information). Fe‐MOF nanocrystals showed almost the same crystal cell parameters as FeCl_3_‐MOF nanocrystals, possibly due to the spatial neutralization between chlorine elimination (Figure S3, Supporting Information) and reduction‐induced enlargement of Fe diameter. It was found that the reduction processes from FeCl_3_‐MOF to Fe‐MOF had almost no influence on the structure and morphology of MOF, indicating their excellent stability in structure favoring steady drug delivery.

From the absorption spectra (Figure 1f), a visible redshift of the Soret band of porphyrin from 417 to 452 nm after coordination with Fe can be ascribed to strong electronic coupling between porphyrin rings. Surprisingly, the absorbances of various bands of Fe‐MOF nanocrystals were markedly lower than that of FeCl_3_‐MOF nanocrystals at the same molar concentration, possibly due to the neutralization between the electrophilic effect of pyridine and the electrophobic effect of Fe(0) or/and the enhanced coordination attraction between pyridine and Fe after Fe reduction. This was also possibly the reason causing the difference in Fourier transform infrared (FTIR) spectra between FeCl_3_‐MOF and Fe‐MOF (Figure S4, Supporting Information). Furthermore, FeCl_3_‐MOF and Fe‐MOF nanocrystals exhibited a similar but different fluorescence feature in comparison with TPyP (Figure 1g and Figure S5, Supporting Information) and the fluorescence intensity became weaker and weaker with Fe coordination and reduction. The fluorescence property of Fe‐MOF nanocrystals enabled in vitro and in vivo tracing by fluorescence imaging. From N_2_ adsorption‒desorption isotherms and the corresponding distributions of pore size (Figure S6, Supporting Information), the specific surface area and pore volume of Fe‐MOF nanocrystals decreased from 117 m^2^ g^−1^ and 0.35 cm^3^ g^−1^ to 103.8 m^2^ g^−1^ and 0.25 cm^3^ g^−1^, respectively, after DOX loading. The decreases in pore volume and specific surface area indicated successful loading of DOX into the pore channels of Fe‐MOF nanocrystals (DOX@Fe‐MOF). The DOX loading capacity of Fe‐MOF was measured to be as high as 940 mg g^−1^ (Figure S7, Supporting Information), possibly owing to the microporous feature of Fe‐MOF and the hydrogen bonds between pyridyl groups of Fe‐MOF and amino/hydroxyl groups of DOX. From the adsorption spectrum of DOX@Fe‐MOF (Figure S8, Supporting Information), the characteristic peak of DOX at 480 nm was obvious, indicating the successful encapsulation of DOX into DOX@Fe‐MOF. From DLS, TEM, and XRD data (Figures S1, S9, and S10, Supporting Information), the DOX loading had not changed the structure and morphology of Fe‐MOF nanocrystals. The attachment stability of DOX into the Fe‐MOF was investigated in vitro by measuring the UV absorption spectra of DOX released from DOX@Fe‐MOF in phosphate buffered solution, fetal bovine serum (FBS) and mouse serum for 24 h, and the corresponding DOX releasing amounts were calculated to be only 1.44%, 1.55%, and 1.58% according to the Lambert‐Beer law (Figure S11, Supporting Information). In addition, the hemolysis assay was also applied to assess the stability of the attached DOX into the Fe‐MOF. The hemolysis ratios of all investigated Fe‐MOF and DOX@Fe‐MOF samples were no more than 5% (Figures S12 and S13, Supporting Information). These results consistently indicated that the leakage of DOX from DOX@Fe‐MOF was not obvious.

### 2.2 Acid‐Responsive Degradation and Hydrogen/Drug Release Profiles

**Figure 2:**
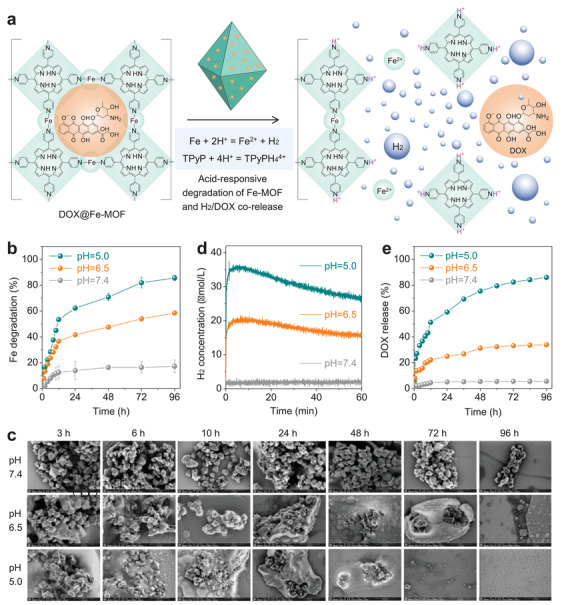
Acid‐responsive degradation and hydrogen/drug release profiles. Schematic illustration of acid‐controlled hydrogen/drug release from a) DOX@Fe‐MOF, b,c) the degradability, d) H_2_ releasing behaviors, and e) DOX releasing profiles of Fe‐MOF nanocrystals in PBS at different pH values.

The biodegradation of drug carriers is of great significance for biosafety. One of the merits of designing Fe‐MOF nanocrystals as drug carrier is that the coordination between Fe(0) and TPyP is able to disassemble after acid‐responsive degradation of Fe(0), endowing Fe‐MOF with acid‐responsive degradation. Another merit is the utilization of porosity and degradation of Fe‐MOF nanocrystals for degradation‐mediated corelease of loaded drug (**Figure** **2**a). Therefore, the acid‐responsive degradation and drug release behaviors of Fe‐MOF nanocrystals were investigated at the same time. Released free Fe ions (Figure 2b) and free DOX (Figure 2e) were collected by filtration to be determined by inductively coupled plasma atomic emission spectrometer (ICP‐AES) and ultraviolet and visible spectrophotometer (UV–vis), respectively, while generated hydrogen molecules (Figure 2d) were detected in real time by a hydrogen microelectrode. As depicted in Figure 2b, Fe‐MOF nanocrystals sharply disassembled into free ions in the acidic phosphate buffer saline (PBS) environment and lower pH value of PBS caused Fe‐MOF nanocrystals faster degraded, which was further verified by the gradual losses in octahedron morphology, nanoscale size, high dispersion and solid mass (Figure 2c). Correspondingly, both hydrogen gas and the loaded drug DOX were sensibly generated in an acid‐responsive way (Figure 2d,e). It seems that Fe‐MOF nanocrystals also degraded in pH = 7.4 PBS slowly (Figure 2b and Figure S14, Supporting Information), possibly owing to high activity of single‐atom Fe(0), but it is worth noting that Fe‐MOF nanocrystals can still maintain stable in the first one hour without visible degradation (Figure 2a), hydrogen generation (Figure 2d) and drug leakage (Figure 2e). Such a time window is long enough for intratumoral accumulation of DOX@Fe‐MOF nanocrystals after intravenous injection. For a long‐term incubation in pH = 7.4 PBS, Fe‐MOF nanocrystals were indeed able to degrade into metabolizable ions (TPyP and Fe ions), and subsequently hydrogen gas and DOX were released slowly (Figure 2b, Figure S14, Supporting Information, and Figure 2e). Noticeably, quick drug release in the acidic environment of tumor (pH = 6.5) can favor to kill tumor cells while slow drug release in normal tissues (pH = 7.4) can avoid acute toxicity of drug to normal cells/tissues and hydrogen gas can not only enhance anticancer effect of chemotherapeutic drug but also attenuate toxic side effects of chemotherapeutic drug.^[^ ^25^ ^]^ It can be found that by virtue of Fe‐MOF nanocrystals, the conflict between carrier degradation/metabolism and drug leakage has been well addressed, and the characteristic of generating hydrogen molecules with a unique cell‐selectivity ingeniously compensates for avoidable off‐target‐caused toxic side effects of chemotherapeutic drugs, implying relatively high biosafety in favor of clinical translation.

### 2.3 In Vitro Hydrogen‐Chemotherapy Efficacies and Mechanisms

First, the drug resistance of used the DOX resistant human breast cancer (MCF‐7/ADR) cells was checked. From Figure S15A in the Supporting Information, the resistant index was calculated to be 12.6, which was above ten times compared to human breast cancer (MCF‐7) cells, indicating the qualified multidrug resistance (MDR) feature of used MCF‐7/ADR cells. We further performed RNA‐seq and western blot (WB) to compare the difference of P‐gp protein expression between MCF‐7 cells and MCF‐7/ADR cells. As shown in Figure S15B in the Supporting Information, the level of P‐gp protein in MCF‐7/ADR cells was remarkably higher than that in MCF‐7 cells. In addition, verapamil and rhodamine 123 were used as a P‐gp inhibitor and a substrate of P‐gp, respectively, to check the drug efflux capability of MCF‐7/ADR cells. From Figure S15C in the Supporting Information, rhodamine 123 was hardly accumulated in MCF‐7/ADR cells compared to parental MCF‐7 cells, but the rhodamine 123 accumulation in MCF‐7/ADR cells was restored to approximate the level in MCF‐7 cells after P‐gp was inhibited by verapamil, suggesting that high P‐gp protein expression in MCF‐7/ADR cells played an efficient function of MDR. Finally, MCF‐7 and MCF‐7/ADR cells were further evaluated by transcriptomic analysis (Figure S16, Supporting Information). From the volcano plot in Figure S15A in the Supporting Information, 19.3% and 18.3% genes were respectively upregulated and downregulated in MCF‐7/ADR cells compared to MCF‐7 cells, indicating the remarkable difference between MCF‐7/ADR cells and MCF‐7 cells. From the kyoto encyclopedin of gene and genomes (KEGG) pathway (human diseases) enrichment analysis of differentially expressed genes in Figure S16B in the Supporting Information, there were 98 differentially expressed genes related to drug resistance. From the gene ontology (GO) enrichment analysis in Figure S16C in the Supporting Information, 95 genes enriched significantly in cancer and drug resistance processes, indicating that MCF‐7/ADR cells were indeed drug‐resistant breast cancer cells. From the heat map of drug resistance related genes between MCF‐7/ADR cells and MCF‐7 cells in Figure S16E in the Supporting Information demonstrated the drug resistance property of MCF‐7/ADR cells. Therefore, MCF‐7/ADR cells could be used as an MDR cell model.

**Figure 3:**
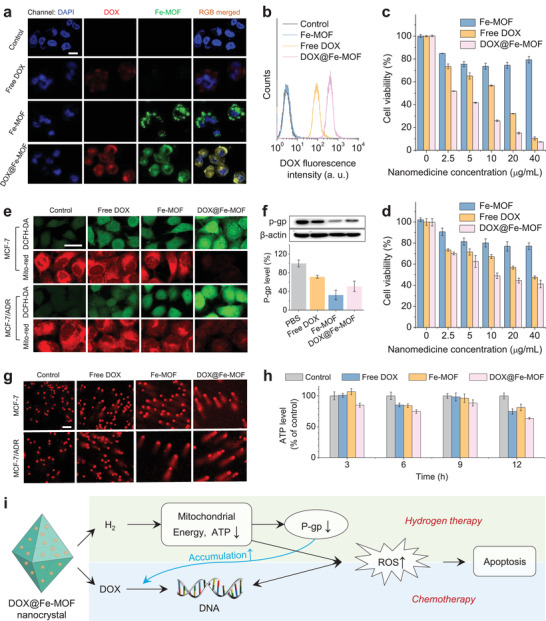
In vitro combined hydrogen‐chemotherapy efficacies and mechanisms for overcoming cancer MDR. a,b) The cellular uptake behavior of nanomedicine by MCF‐7/ADR cells after incubation for 4 h, the cytotoxicity of nanocrystals against c) MCF‐7 and d) MCF‐7/ADR cells (*n* = 4 independent experiments), e) confocal images of ROS (green fluorescence) and mitochondrial membrane potential (red fluorescence), for f) the intracellular level of P‐gp by western blot measurement (*n* = 3 independent experiments), g) DNA damage of cancer cells, h) intracellular ATP level in treated MCF‐7/ADR cells, and i) schematic illustration of combined hydrogen‐chemotherapy mechanism. All scale bars correspond to 30 µm.

Cellular uptake behavior of the nanocrystals was investigated first. As depicted in Figure 1g, Fe‐MOF and FeCl_3_‐MOF nanocrystals could be tracked by fluorescence imaging due to their inherent fluorescence property. From **Figure** **3**a and Figure S17a in the Supporting Information, it can be seen that the nanocrystals were largely endocytosed by both MCF‐7 cells (Figure S17a, Supporting Information) and MCF‐7/ADR cells (Figure 3a) after 4 h incubation. Different to MCF‐7 cells (Figure S17b, Supporting Information), the intracellular red/DOX fluorescence intensity from DOX@Fe‐MOF in MCF‐7/ADR cells was much higher than that of free DOX by flow cytometry analysis (Figure 3b), suggesting that the nanocrystals‐mediated endocytosis or/and nanocrystals‐generating hydrogen gas increased the DOX uptaking capability of MCF‐7/ADR cells. Furthermore, the in vitro anticancer performances of the DOX@Fe‐MOF nanomedicine were investigated using several cell lines. As shown in Figure 3c, the cytotoxicity of Fe‐MOF against MCF‐7 cells was weak, suggesting that the anticancer effect of hydrogen gas was slight. However, DOX@Fe‐MOF exhibited remarkably higher cytotoxicity than that of both free DOX and DOX@Fe‐MOF at the same molar concentration in a concentration‐dependent manner even though DOX release from DOX@Fe‐MOF was sustained (Figure S18, Supporting Information), which indicated that H_2_ enhanced the anticancer efficacy of DOX. In addition, longer treatment time with DOX@Fe‐MOF caused higher cytotoxicity to both MCF‐7 and MCF‐7/ADR cells (Figure S19, Supporting Information). Moreover, the chemotherapeutic sensitization effect of hydrogen gas occurred in various types of cancer cells, including human cervical carcinoma (HeLa) (Figure S20a, Supporting Information), lung cancer cells (A549) (Figure S20b, Supporting Information), mouse breast cancer cells (4T1) cells (Figure S20c, Supporting Information), and even MCF‐7/ADR cells (Figure 3d). This sensitization effect was more significant in MDR cells as chemotherapy was quite limited (Figure 3d).

**Figure 4:**
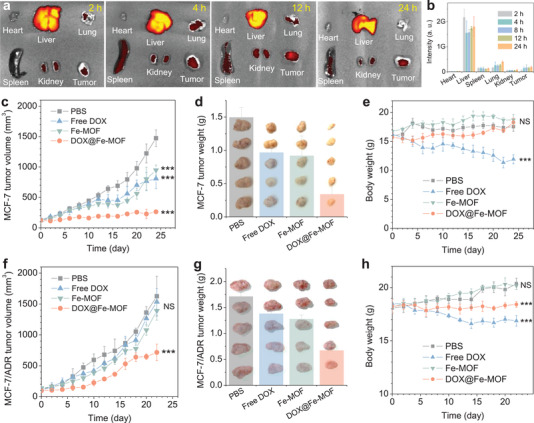
In vivo combined hydrogen‐chemotherapy (*n* = 5 independent experiments). a) Fluorescence images of organs extracted from tumor‐bearing mice after intravenous injection with Fe‐MOF nanocrystals for different time periods and b) the corresponding fluorescence intensity, c–e) the outcomes of MCF‐7 and f–h) MCF‐7/ADR tumor therapy with Fe‐MOF nanocrystals: c,f) the tumor volume change with time, d,g) photographs of tumors extracted at the end of treatment, and e,h) the body weight change during treatment. *P < 0.05, **P < 0.01, ***P < 0.001.

The commonest mechanism in MDR involves the efflux of drugs from MDR cells mediated by adenosine triphosphate (ATP)‐binding cassette transporters, which bind ATP and then utilize the energy from ATP hydrolysis to drive drug transportation.^[^ ^32^ ^]^ Recent research has strongly prioritized the suppression of P‐glycoprotein (P‐gp, an efflux pump protein) expression to increase intracellular drug accumulation for the mitigation of MDR.^[^ ^33^ ^]^ Since hydrogen gas has proven to be an excellent mitochondria‐targeted energy regulator capable of regulating the energy of diseased cells,^[^ ^35^, ^36^ ^]^ we here hypothesized that hydrogen gas could suppress P‐gp expression by downregulating the energy of MDR cells and intracellular ATP level for anti‐MDR, which is one of the main reasons why we integrated hydrogen therapy with chemotherapy through Fe‐MOF nanocrystals in this work. As shown by the intracellular intensity of red fluorescence, Fe‐MOF slightly decreased the mitochondrial membrane potential of MCF‐7 (Figure 3e), MCF‐7/ADR (Figure 3e) and 4T1 cells (Figure S21a, Supporting Information), suggesting the weak damage of hydrogen gas to the function of mitochondria in cancer cells which can depress intracellular ATP expression and P‐gp activity. By comparison, combined hydrogen‐chemotherapy by DOX@Fe‐MOF markedly damaged the function of mitochondria. Correspondingly, the levels of ATP and P‐gp were inhibited more by DOX@Fe‐MOF than by free DOX and by Fe‐MOF (Figure 3f,h). Therefore, the released DOX from DOX@Fe‐MOF was effectively accumulated in MCF‐7/ADR cells (Figure S22, Supporting Information), favoring enhanced anti‐MDR efficacy (Figure 3d). Furthermore, intracellular reactive oxygen species (ROS) level in MCF‐7 and MCF‐7/ADR cells was enhanced both by free DOX and by Fe‐MOF (Figure 3e), which caused no further damage by hydrogen gas to DOX‐damaged DNA (Figure 3g and Figure S21b,c, Supporting Information). By comparison, DOX@Fe‐MOF exhibited the remarkable enhancement of intracellular ROS level (Figure 3e, Figures S21b and S22, Supporting Information), leading to increased anticancer efficacy of combined hydrogen‐chemotherapy (Figure 3c,d). Thus, combined hydrogen‐chemotherapy exhibited enhanced anti‐MDR efficacies by energy‐mediated P‐gp downregulation and ROS‐mediated DNA damage, as schematically illustrated in Figure 3i.

### 2.4 In Vivo Hydrogen‐Chemotherapy Outcomes

Passive tumor‐targeted delivery of Fe‐MOF nanocrystals was first checked firstly since their size was small. The biodistribution of nanomedicines can generally be quantified by elemental analysis with the ICP‐AES technique, but tissues and blood contained large amounts of Fe, which makes ICP‐AES method unsuitable for the determination of Fe‐MOF biodistribution. Fortunately, Fe‐MOF nanocrystals have a unique fluorescence feature (Figure 1g), which can be used to semi‐quantify their biodistribution by fluorescence imaging. From **Figure** **4**a,b, it can be seen that Fe‐MOF nanocrystals steadily accumulated in tumors after intravenous injection into MCF‐7 tumor‐bearing mice. The pharmacokinetic experiment of DOX@Fe‐MOF indicated that its half‐time of blood circulation was about 1.7 h in favor of its intratumoral accumulation (Figure S23, Supporting Information). Subsequently, the anticancer efficacy of DOX@Fe‐MOF nanomedicine after intravenous injection (5 mg kg^−1^) was investigated in MCF‐7 and MCF‐7/ADR tumor‐bearing mouse models. MCF‐7 and MCF‐7/ADR tumor‐bearing mice were randomly divided into four groups (*n *= 5) to evaluate the therapeutic effect in vivo. As demonstrated in Figure 4c,d, both free DOX and Fe‐MOF inhibited MCF‐7 tumor growth slightly, which meant that both chemotherapy and hydrogen therapy can eliminate tumors to a certain extent. By comparison, the growth of MCF‐7 tumors was efficiently inhibited, suggesting augmented anticancer efficacy of combined hydrogen‐chemotherapy, in accordance with the in vitro results (Figure 3c). Furthermore, MCF‐7/ADR tumors were more insensitive to free DOX and Fe‐MOF than MCF‐7 tumors (Figure 4f,g). Even so, the combined effect of hydrogen‐chemotherapy with DOX@Fe‐MOF nanomedicine still worked on MCF‐7/ADR tumors (Figure 4f,g) in accordance with the in vitro results (Figure 3d), which was further confirmed by the TdT‐mediated dUTP Nick‐End labeling (TUNEL) assay of tumor tissues at the end of treatment (Figure S24, Supporting Information). The MCF‐7/ADR tumor inhibition rate of DOX@Fe‐MOF reached 60.7% after 22 d treatment (Figure 4f,g), which indicated that hydrogen gas could efficiently sensitize chemotherapy to overcome cancer MDR. Most impressively, in contrast to individual chemotherapy, combined hydrogen‐chemotherapy did not cause obvious body weight loss in MCF‐7 and MCF‐7/ADR tumor‐bearing mice during treatment (Figure 4e,h), although tumor volumes/weights gradually decreased (Figure 4c,d,f,g), implying that hydrogen gas could reduce the toxic side effects of chemotherapy.

Antimetastasis is much more difficult than antiprimary tumor as reflected by the lethality of metastases and higher mortality of metastatic patients.^[^ ^,37^ ^]^ MMPs have been proven to play an important role in promoting metastasis by proteolysis of the extracellular matrix and by the activation of signaling pathways of tumor cell migration.^[^ ^38^ ^]^ Unexpectedly, we revealed that hydrogen therapy with Fe‐MOF nanocrystals can distinctly downregulate the expression of MMP‐2 in 4T1 breast cancer and combined hydrogen‐chemotherapy with DOX@Fe‐MOF nanocrystals more markedly inhibited the expression of MMP‐2 (Figure S25, Supporting Information). Therefore, we hypothesized that combined hydrogen‐chemotherapy with DOX@Fe‐MOF nanocrystals could block the metastasis of primary 4T1 breast cancer. A lung metastasis model was built with 4T1‐Luciferase (4T1‐LUC) breast cancer cells which were labeled with fluorescence for real‐time monitoring of tumor growth and metastasis by in vivo fluorescence imaging.

**Figure 5:**
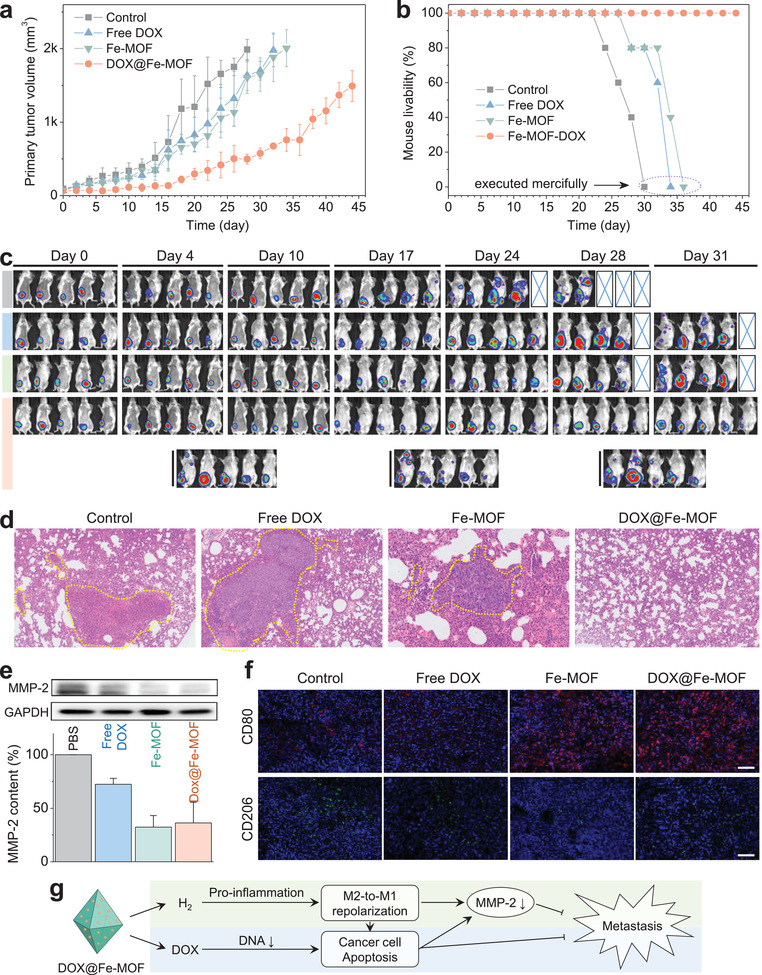
Inhibition of the growth and lung metastasis of primary breast tumor by DOX@Fe‐MOF nanomedicine. a) The volume change in primary 4T1 tumors during treatment (*n* = 5 independent experiments), b) the survival rate of 4T1 tumor‐bearing mice during treatment (*n* = 5 independent experiments), c) the fluorescence tracking of 4T1‐LUC tumor growth and metastasis, and d) histological analysis of the lung by H&E staining, e) intratumoral expression of MMP‐2 detected by WB where glyceraldehyde‐3‐phosphate dehydrogenase (GAPDH) was a loading control (*n* = 3 independent experiments), f) intratumoral expression of M1/M2 macrophages‐specific proteins CD80 and CD206 after treatment, and g) schematic illustration of combined hydrogen‐chemotherapy mechanism for antimetastasis. The scale bars in (f) correspond to 100 µm.

As shown in **Figure** **5**a–c, the treatment outcome of primary 4T1 tumors was similar to that of MCF‐7 and MCF‐7/ADR tumors as mentioned above. The 4T1 tumor inhibition effects of free DOX and Fe‐MOF were not obvious, while that of DOX@Fe‐MOF was much more markedly, indicating that combined hydrogen‐chemotherapy more efficiently inhibited the growth of primary 4T1 tumors compared with individual chemotherapy and hydrogen therapy. Moreover, the survival time of 4T1 tumor‐bearing mice was more significantly prolonged (at least 50−87.5% elongation) by combined hydrogen‐chemotherapy (Figure 5b). All 4T1 tumor‐bearing mice in the PBS control group died after 30 d, while all 4T1 tumor‐bearing mice treated with DOX@Fe‐MOF nanocrystals survived up to 45 d. In addition, in vivo real‐time fluorescence tracking results indicated that tumor‐bearing mice in the PBS control group developed lung metastasis after about 17 d and quickly died after 30 d, and in both the free DOX and Fe‐MOF groups the time to tumor metastasis was prolonged ≈28 d (64.7% increase) and mouse survival increased to ≈34 d (13.3% increase), respectively (Figure 5c). By comparison, DOX@Fe‐MOF inhibited lung metastasis time by 111.8%, and only 60% of 4T1 tumor‐bearing mice developed lung metastasis after treatment for 44 d with a 158.8% increase in lung metastasis time while another 40% of mice did not experience lung metastasis (Figure 5c). This indicated that combined hydrogen‐chemotherapy could efficiently inhibit lung metastasis. Furthermore, lung histological analysis suggested that metastatic tumors in the lung generally existed in the free DOX and Fe‐MOF groups, but lung tissue in surviving mice without lung metastasis in the DOX@Fe‐MOF group did not demonstrate visible tumor or pulmonary nodules (Figure 5d and Figures S26 and S27, Supporting Information), further confirming the excellent antimetastasis effect of combined hydrogen‐chemotherapy. In addition, the inhibition of primary tumor growth probably made contribution to decreased metastasis to a certain extent. But it was hard to separate the killing effect of DOX@Fe‐MOF on primary tumors from its metastasis‐inhibiting effect for determining the proportion of this contribution.

To investigate the mechanism of antimetastasis, the expression of MMP‐2 in tumor was measured. From Figure 5e, Fe‐MOF nanocrystals markedly depressed the intratumoral expression of MMP‐2 in accordance to the in vitro results (Figure S25, Supporting Information). Both cancer cells and TAMs possibly made certain contribution to the depressed expression of MMP‐2 in tumor. As well known, M2‐phenotype tumor‐related macrophages (TAMs) help cancer cells metastasis by secreting MMP‐2, while M1 phenotype is tumoricidal.^[^ ^40^, ^41^, ^42^ ^]^ Therefore, we further investigated the effect of Fe‐MOF on the phenotype of TAMs. From fluorescence images of extracted tumors in Figure 5f where red and green fluorescences represented M1 and M2 phenotypes of macrophages, respectively, we unexpectedly discovered that Fe‐MOF nanocrystals clearly induced the M2‐to‐M1 polarization of intratumoral macrophages, which was possibly the partial reason why Fe‐MOF nanocrystals depressed the intratumoral expression of MMP‐2. In free DOX group, the amount of both M2‐phenotype and M1‐phenotype macrophages decreased, owing to the cytotoxicity of DOX. Moreover, M1‐phenotype macrophages can kill cancer cells locally. In addition, we further confirmed Fe‐MOF nanocrystals can induce the M2‐to‐M1 polarization of RAW264.7 macrophages in vitro (Figure S28, Supporting Information), and even can effectively reverse the interleukin 4 (IL‐4)‐induced M2‐like macrophages to the M1 phenotype to a certain extent (Figure S29, Supporting Information). Thus, we can deduct that hydrogen molecule released from Fe‐MOF nanocrystals played a role as immunoactivator^[^ ^42^ ^]^ in making contribution to antimetastasis by inducing the M2‐to‐M1 transformation of TAMs and consequently downregulating the expression of MMP‐2, as illustrated in Figure 5g. On the other hand, DOX also inhibited the intratumoral level of MMP‐2 (Figure 5e) and cancer metastasis to a certain extent (Figure 5c), possibly owing to induced apoptosis of M2‐phenotype macrophages and cancer cells. By combination of hydrogen‐chemotherapy with DOX@Fe‐MOF, enhanced antimetastasis efficacy was achieved (Figure 5c,g). Although there possibly were other pathways besides MMP‐2 to mediate antimetastasis, we think the DOX@Fe‐MOF‐mediated downregulation of MMP‐2 was an important pathway for antimetastasis from the present data and conclusion.

### 2.5 Biocompatibility and Biosafety Properties

**Figure 6:**
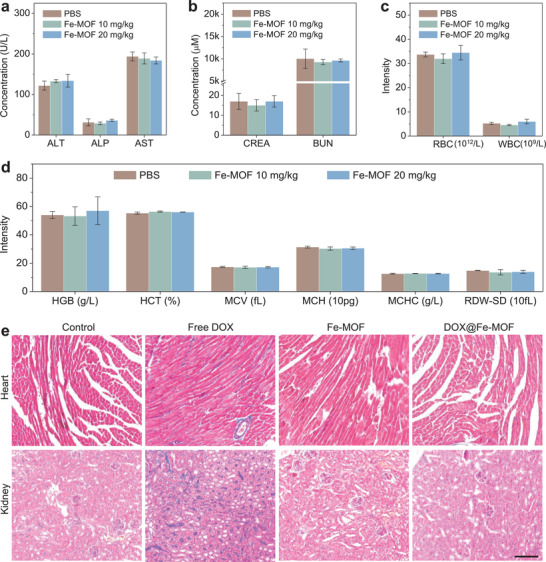
The biocompatibility and biosafety of Fe‐MOF nanocrystals (*n* = 3 independent experiments). Blood biochemical analyses including a) liver and b) kidney functions, c,d) the evaluation of standard hematology markers of mice one week after intravenous injection at different doses, and e) Masson's trichrome staining of the heart and kidney in treated 4T1 tumor‐bearing mice. The scale bar corresponds to 100 µm.

The biosafety of nanomedicines is vitally important to their clinical translation. In this work, we chose two types of materials, porphyrin and Fe, proven for clinical applications by the FDA, to construct Fe‐MOF nanocrystals as both a drug carrier and hydrogen donor in order to obtain high biocompatibility and to endow the constructed DOX@Fe‐MOF nanomedicine with high biosafety by virtue of the chemotherapeutic toxic side effect‐attenuated capability of hydrogen gas. As Fe‐MOF nanocrystals mainly accumulated in the liver, spleen and kidney in addition to the tumor after intravenous injection, and degradation products including TPyP and Fe ions were mainly cleared by the liver and kidney, and the liver and kidney functions were evaluated by several key indicators (alkaline phosphatase, alanine transaminase, aspartate transaminase, creatinine (CREA), blood urea nitrogen) after injection of high doses of Fe‐MOF (10 and 20 mg kg^−1^). First, Fe‐MOF nanocrystals did not exhibit visible cytotoxicity to various normal cells including embryonic fibroblasts (3T3 cells), embryonic kidney cells (293T cells), and human foreskin fibroblasts (HFF cells), indicating high cytocompatibility (Figure S30, Supporting Information). From **Figure** **6**a,b, it can be seen that free DOX caused distinct damage to the liver and kidney at the dose of 2.5 mg kg^−1^ due to its nonspecific cytotoxicity, Fe‐MOF nanocrystals caused no visible toxicity in the liver and kidney of mice even at an eightfold higher dose, indicating that the Fe‐MOF carrier possessed high tissue compatibility. Furthermore, the standard hematology analysis of several main markers (red blood cells (RBC), white blood cells, hemoglobin (HGB), hematocrit, mean corpuscular hemoglobin, mean corpuscular volume, mean corpuscular hemoglobin concentration, red blood cell volume distribution width‐standard deviation (RDW‐SD) was carried out to assess the systematic biocompatibility of Fe‐MOF. From Figure 6c,d, it can be seen that Fe‐MOF exhibited high systematic biocompatibility one week after injection. Moreover, histological analysis of the main organs including heart, liver, spleen, lung, and kidney was investigated by hematoxylin‒eosin (H&E) staining to evaluate tissue compatibility of Fe‐MOF nanocrystals. As shown in Figures S31–S34 in the Supporting Information, Fe‐MOF did not display obvious tissue damages or noticeable pathological changes in three tumors‐bearing models. These results indicated that Fe‐MOF nanocrystals as a drug carrier had high biosafety.

Furthermore, we also assessed whether the Fe‐MOF carrier contributed to the biocompatibility and biosafety of the constructed DOX@Fe‐MOF nanomedicine. The nonspecific toxicity of DOX mainly acts on the heart and kidney and therefore the effects of the DOX@Fe‐MOF nanomedicine on the heart and kidney were investigated. From Figure S35 in the Supporting Information, it can be seen that free DOX caused distinct damage to the kidney at the dose of 2.5 mg kg^−1^ as indicated by a marked decrease in the ratio of UREA/CREA, but the constructed DOX@Fe‐MOF nanomedicine caused no visible toxicity in the kidney of mice at the same dose, suggesting that the Fe‐MOF carrier completely eliminated the toxic side effects of chemotherapeutic drug DOX due to its hydrogen‐generating capability. The histological assessment using Masson's trichrome staining (Figure 6e) showed that free DOX caused severe fibrosis of the heart and kidney, but neither Fe‐MOF nor DOX@Fe‐MOF did, indicating that Fe‐MOF effectively prevented DOX from causing damage to tissues, possibly due to the anti‐inflammatory effect of hydrogen gas released from Fe‐MOF.^[^ ^41^ ^]^ These results suggested that Fe‐MOF not only possessed high biosafety, but can also eradicate the toxic side effects of chemotherapeutic drugs, thus, has great potential for clinical translation.

## 3 Conclusion

In summary, we developed a new type of Fe‐MOF nanocrystals as drug carrier and hydrogen donor for combined hydrogen‐chemotherapy of MDR and metastasis. The constructed DOX@Fe‐MOF nanomedicine exhibited excellent acid‐responsive degradation and hydrogen/drug release behaviors. The released hydrogen gas from the nanomedicine efficiently sensitized the chemotherapy of MCF‐7/ADR cells by downregulating P‐gp expression and decreasing the ATP level for enhancement of ROS‐mediated DNA damage, and assisted DOX to effectively inhibit metastasis by immunoactivating M1 macrophages and suppressing the expression of MMP‐2. On the other hand, the released hydrogen gas from the nanomedicine also reduced the toxic side effects of DOX to normal tissues, endowing the DOX@Fe‐MOF nanomedicine with excellent biocompatibility and biosafety. Both high anti‐MDR and antimetastasis outcomes and high biosafety of the DOX@Fe‐MOF nanomedicine benefited from the excellent versatile Fe‐MOF platform and ensured its high potential for clinical translation.

## 4 Experimental Section

### Chemicals

5,10,15,20‐Tetrakis(4‐pyridyl)‐21H,23H‐porphine (TPyP) and sodium borohydride were purchased from J&K Scientific Ltd (Beijing, China). Polyvinylpyrrolidone (PVP, molecular weight = 55 000 Daltons), iron chloride hexahydrate (FeCl_3_·6H_2_O), and hydrochloric acid (37%) were provided by Sinopharm Chemical Reagent Co., Ltd. DOX was obtained from Shanghai Macklin Biochemical Co., Ltd. The cell counting kit‐8 (CCK‐8) kit was purchased from Beyotime Biotechnology Co., Ltd. All other agents used were of the highest commercial grade available.

### Synthesis and Characterization of Fe‐MOF and DOX@Fe‐MOF

Fe‐TPyP nanocrystals were prepared by a coordination reaction between FeCl_3_ and TPyP and then reduced by sodium borohydride to obtain Fe‐MOF nanocrystals. First, TPyP (12.8 mg, 0.02 mmol) was completely dissolved in 4 mL 10 × 10^−3^ m aqueous solution of hydrochloric acid and then added in 40 mL 1 × 10^−3^ m aqueous solution of FeCl_3_. An aqueous solution of sodium carbonate (2 mL, 100 × 10^−3^ m) was quickly added into the above mixed solution at 50 °C under vigorous stirring. After 4 h, the suspension was collected by centrifugation for 20 min at 14 000 rpm and washed with deionized water three times and resuspended in water. Then, 10 mg PVP was added to the suspension solution for 1‐h ultrasonic vibration to modify and stabilize FeCl_3_‐MOF nanocrystals. Finally, excessive aqueous solution of NaBH_4_ (7.6 mg in 2 mL) was added dropwise under stirring at 4 °C to reduce Fe^3+^ to Fe(0). After 3 h, Fe‐MOF nanocrystals were collected by centrifugation (14 000 r min^−1^) and washed twice with water.

To load DOX, DOX (5 mg mL^−1^) was dissolved in the aqueous solution of Fe‐MOF nanocrystals (5 mg mL^−1^), and then the pH value of the mixed solution was adjusted to 7 by dropwise adding an aqueous solution of NaOH (0.1 m) under stirring. The mixed solution was vacuumized for 15 min and then DOX loaded Fe‐MOF nanocrystals were collected by centrifugation and washed twice with deionized water. The UV absorption of the collected supernatant was measured and the content of DOX in the supernatant was calculated by the Lambert–Beer law according to the standard curve. Finally, the DOX loading capacity of Fe‐MOF can be calculated.

The morphology and structure of Fe‐MOF nanocrystals were measured by a SEM (APREO‐S, FEI) under a low voltage (500**‒**800 V). The crystal phase structure of Fe‐MOF nanocrystals was characterized by powder XRD using an M21X diffractometer (Cu K*α*, *λ* = 1.54056 Å, 40 kV) and SAXS (Xeuss2.0). The hydrated size of Fe‐MOF was detected by DLS (Malvern Nano‐ZS). The composition of Fe‐MOF was measured by attenuated total reflectance (ATR) FTIR spectroscopy on a Thermo‐Nicolet Nexus 670 ATR‐IR spectrometer. UV spectra were recorded on a Genesys 10S UV–vis spectrophotometer (Thermo Scientific) at room temperature. A fluorescence spectrophotometer (Thermo Scientific) was used for fluorescence detection. A Leica TCS SP5II (Germany) confocal laser scanning microscope (CLSM) microscope was used to measure cellular uptake behavior and in vitro hydrogen‐chemotherapy mechanisms. The in vivo fluorescence imaging was conducted on an IVIS LuminaII + XGI‐8 (gas anesthesia system) from Caliper Life Science.

### Measurement of Acid‐Responsive Degradation and Hydrogen/Drug Corelease

For the degradation study, Fe‐MOF nanocrystals were dispersed in PBS at various pH values (pH = 5.0, 6.5, and 7.4) at 37 °C and the supernatant was collected by filtration using a 14 000 Dalton dialysis bag at different time points after shaking. The concentration of released Fe ions in the collected supernatant was measured using inductively coupled plasma atomic emission spectrometer (ICP‐AES).

To investigate DOX release, 1 mg DOX@Fe‐MOF nanomedicine was suspended in 1.8 mL PBS at pH 5.0, 6.5 or pH 7.4 and shaken in the dark. After a specified time interval, the supernatant was collected by filtration using a 14 000 Dalton dialysis bag and measured by UV–vis to determine the amount of DOX released. With regard to acid‐responsive hydrogen release, hydrogen concentration was quantitatively monitored in real time using a hydrogen microelectrode (Unisense, Denmark).

### Hemolysis Assay

Hemolysis was one of the most important concerns regarding its safety when administrating drug loaded nanomedicine into the body. MRBCs were obtained from two Balb/c mouse and isolated by centrifugation from removing excess plasma for several times. The diluted RBC suspension was added to Fe‐MOF and DOX@Fe‐MOF solutions at a sequence of concentrations (from 12.5 to 100 µg mL^−1^). After placing in the dark at room temperature for 3 h, the samples were centrifuged and the supernatant was taken for absorbance measurement (Abs 576 nm). The percent hemolysis of the RBCs was calculated using the formula reported in a previous study.

### Cell Culture and Mice

Human breast cancer MCF‐7 and MCF‐7/ADR cells were purchased from Shanghai Meixuan Biotechnology Co., Ltd. (China), and mouse breast cancer cells of 4T1 and 4T1‐LUC, mouse embryonic fibroblasts (3T3 cells), mouse embryonic kidney cells (293T cells), and HFF cells were purchased from China Type Culture Collection obtained from the American Type Culture Collection. These cells were cultured in Dulbecco minimum essential medium containing 10% v/v FBS, penicillin (100 U mL^−1^), and streptomycin (100 mg mL^−1^) in a humidified incubation chamber at 37 °C and 5% CO_2_.

Female Balb/c nude mice were purchased from Shanghai BiKai (BK Lab), and raised under standard conditions at 25 ± 2 °C and 60% ± 10% humidity in a 12 h light/12 h dark cycle. Animal care and handling procedures were in agreement with the guidelines evaluated and approved by the Animal Experiment Ethics Committee of Fudan University (2014‐03‐YJ‐PZQ‐01).

### Measurement of Cellular Uptake of Nanocrystals

For CLSM imaging, MCF‐7 and MCF‐7/ADR cells were seeded in 35 mm glass‐bottomed dishes, respectively, and cultured for 12 h, and then the DOX@Fe‐MOF nanomedicine (500 µL, 20 µg mL^−1^) was added. After culture for 4 h at 37 °C, the culture medium was removed, and the nanoparticles outside cells were removed by washing with PBS three times. Then, 1 mL anhydrous methanol solution of 4′,6‐diamidino‐2‐phenylindole (1.5 µg mL^−1^) was added to stain the nuclei for 15 min at 37 °C. The cells were washed with PBS three times to remove residual nanoparticles. Finally, the cells were observed using a CLSM. For flow cytometry analysis, MCF‐7 and MCF‐7/ADR cells were seeded in 6‐well plates and Fe‐MOF nanoparticles (500 µL, 20 µg mL^−1^) were added after incubation for 12 h. After culture for another 4 h, the culture medium was removed, and cells were washed three times with PBS. The cells were then digested by pancreatic enzymes for 3 min and analyzed by flow cytometry (Gallios).

### Detection of Intracellular ROS and ATP levels and DNA and Mitochondrial Damage

For ROS measurement, MCF‐7 and MCF‐7/ADR cells were seeded into 35 mm glass‐bottomed dishes at a density of 5 × 10^4^ cells per dish, and then DOX@Fe‐MOF nanoparticles (500 µL, 20 µg mL^−1^) were added. All the cells were then stained with 10 × 10^−6^ m 2′,7′‐dichlorofluorescin diacetate (DCFH‐DA, an ROS probe, Ex: 488 nm, Em: 537 nm). Fluorescence images of cells were captured using CLSM. For flow cytometry measurement, MCF‐7 and MCF‐7/ADR cells were seeded in six‐well plates and DOX@Fe‐MOF nanoparticles (500 µL, 20 µg mL^−1^) were added. After culture for 4 h, the cells were stained with 10 × 10^−6^ m DCFH‐DA, washed with PBS, digested by pancreatic enzymes for 3 min, and then analyzed by flow cytometry (Gallios). Similarly, after incubation with nanoparticles, 4T1 cells were lysed and the supernatant was immediately collected by centrifugation for the detection of ATP level using an ATP assay kit (Beyotime Biotech.).

To investigate DNA and mitochondrial damage, MCF‐7, MCF‐7/ADR, and 4T1 cells were cultured in a nanoparticle‐containing medium (500 µL, 20 µg mL^−1^) for 12 h, and then stained with MitoTracker Red CMXRos (Beyotime Biotechnology). After incubation for another 20 min, excessive dye was removed by washing with fresh medium, and the cells were then observed using a CLSM (Zeiss LSM880). Nanoparticle‐treated cells were digested by trypsin and washed with PBS, and then intracellular DNA damage was detected by the comet assay.^[^^]^

### Rhodamine 123 Accumulation Assay

The P‐gp activity was determined by measuring the intracellular accumulation of rhodamine 123 in MCF‐7 and MCF7/ADR cells in the absence or presence of P‐gp inhibitors. Briefly, cells were incubated at 37 °C with rhodamine 123 (5.25 × 10^−6^ m) for 30 min, in the presence or absence of various concentrations of verapamil (P‐gp inhibitor, 50 × 10^−6^ m). After washing in phosphate‐buffered saline, cells were lysed in distilled water, and the intracellular level of rhodamine 123 was quantified by spectrofluorimetry using a multimode microplate reader (excitation and emission wavelengths were set to be 485 and 535 nm, respectively).

### Measurement of P‐gp and MMPs Levels

To determine the expression levels of P‐gp and MMP9, MCF‐7, MCF‐7/ADR and 4T1 cells were seeded into six‐well plates, cultured for 24 h, and then the nanoparticles were added. After culture for 12 h, the cells were washed three times with PBS to remove external nanoparticles, and then lysed in an ice bath with the lysis buffer containing 1% protease and phosphatase inhibitors (Sangon Biotech, Shanghai, China). After centrifugation of the lysates at 12 000 rpm for 10 min, the supernatants were collected for detection of protein concentrations using by the bicinchoninic acid (BCA) method (Bio‐Rad, Hercules, CA, USA). The samples were electrophoresed on 8% sodium dodecyl sulfate polyacrylamide gels, and transferred to polyvinylidene difluoride membranes (Millipore, Billerica, MA, USA). After blocking with skimmed milk in Tris buffered saline containing 0.1% Tween‐20 (TBST, pH 7.6) for 1 h, the membranes were incubated overnight with rabbit anti‐P‐gp antibody (1:1000, UK), anti‐MMP‐2 (1:1000, UK)at 4 °C. After washing three times with TBST, the membranes were further incubated with horseradish peroxidase conjugated secondary antibodies for 1 h. The protein bands of interest were detected after incubation with a chemiluminescence substrate (Millipore) using an Amersham Image 600 system (GE, USA). The expression level of MMP‐2 in 4T1 tumors was measured by a similar process. First, the extracted tumors were ground and lysed in an ice bath with lysis buffer to collect the proteins. Then, the samples were electrophoresed on 8% sodium dodecyl sulfate polyacrylamide gels and the expression level of MMP‐2 was measured.

### Measurement of the Polarization of Macrophages In Vivo and In Vitro

Tumor tissues were excised for immunofluorescence staining after tumor‐bearing mice were treated with PBS, free DOX, Fe‐MOF and DOX@Fe‐MOF. Briefly, after blocking with 5% bovine serum albumin (BSA) for 0.5 h at 37 °C, the tumor sections were incubated overnight at 4 °C with anti‐CD80 antibody or anti‐CD206 antibody (Abcam, UK) to label the M1‐like and M2‐like macrophages, respectively. After labeling with secondary antibody, the tumor sections were observed under CLSM.

With regard to in vitro polarization, raw 264.7 macrophages were incubated with lipopolysaccharide + murine interferon (IFN)‐gamma (IFN‐*γ*) for 24 h (M1 polarization), or with interleukin‐4 (IL‐4) for 24 h (M2 polarization) followed by a further 24 h incubation with Fe‐MOF (500 µL, 20 µg mL^−1^) (M1‐to‐M2 repolarization), and then cellular morphology was observed under the microscope. Raw 264.7 macrophages before polarization treatment were used as controls. For the cytofluorescence assay, treated cells were fixed for 15 min with 4% paraformaldehyde, and then labeled with fluoresceine isothiocyanate (FITC) antimouse CD206 antibody or phycoerythrin (PE) antimouse CD80 antibody (BioLegend), and Alexa Fluor 647 antimouse F4/80 antibody in turn for 30 min, followed by flow cytometry analysis (Gallios).

### In Vitro Cytotoxicity Assessment

The cytotoxicity of nanoparticles against MCF‐7, MCF‐7/ADR, 4T1, A549, HeLa, 3T3, HFF, and 293T cells was assessed by the standard CCK‐8 method. Cells were seeded into a 96‐well plate at a density of 5 × 10^4^ per well and then treated with nanoparticles (0, 2.5, 5, 10, 20, and 40 µg mL^−1^). After incubation for another 24 h, CCK‐8 solution was then added to each well and incubated for 1.5 h. Subsequently, the 96‐well plates were measured using an enzymelinked immunosorbent assay reader (Epoch 2 Microplate Spectrophotometer, BioTek Instruments, Inc., USA) at 450 nm. Cytotoxicity was expressed as the percentage of viable cells compared with untreated control cells.

### Pharmacokinetics Studies

For pharmacokinetic study, three Balb/c nude mice (male, 25–27 g) were injected with 100 mL of DOX@Fe‐MOF (1 mg mL^−1^) intravenously. At different time points post injection (0.25, 0.5, 1, 2, 4, 6, 12, 24, and 48 h), 30 µL of blood were collected from mouse orbit and diluted to 970 µL with PBS buffer. Then, diluted blood was added into 96‐well plate for fluorescence intensity measurement using a multimode microplate reader (excitation and emission wavelengths were 452 and 710 nm, respectively).

### Measurement of the Biodistribution of Nanoparticles

MCF‐7 tumor‐bearing BALB/c mice were injected with Fe‐MOF nanoparticle es (1 mg mL^−1^, 100 µL) via the tail vein, then humanly sacrificed at different time points after injection, and imaged on an IVIS spectrum system (Ex = 465 nm, Em = 700 nm). In addition, the tumors and main organs including kidney, lung, spleen, liver and heart were extracted for fluorescence imaging. The biodistribution of nanoparticles was determined by the fluorescence intensity on unit area of extracted organs.

### In Vivo Tumor Therapy Assessment

When the MCF‐7 and MCF‐7/ADR tumors in BALB/c mice grew to about 100 mm^3^, the mice were randomly divided into the following four groups (*n* = 5): the blank control group with PBS, free DOX group, Fe‐MOF group, and DOX@Fe‐MOF group. The injected dose of nanoparticle was 5 mg kg^−1^ body weight. The drug loading of DOX was about 940 mg g^−1^. Therefore, DOX dose in vivo therapy was 2.4 mg kg^−1^ body weight every time. The first nanomedicine therapy day was named as Day 0. Treatment was carried out on day 0, 3, 6, and 9. The body weight of mice and tumor volume were recorded every other day. The tumor volume was determined using the formula (*V* = *a* ^2^ × *b*/2; where *V*, *a*, and *b* represent the tumor volume, and maximum and minimum diameters of tumors, respectively). After treatment, the mice were sacrificed, and their organs (kidney, lung, spleen, liver, heart, and tumor) were extracted and then immersed in 4% paraformaldehyde. Finally, these tissues were cut into slices and stained with H&E for histological analysis. In addition, apoptotic cells in the tumor slices were identified by the TUNEL assay.

For the metastatic 4T1 orthotopic murine breast cancer model, 5 × 10^6^ 4T1‐LUC cells were injected into the breast of each BALB/c mouse. 4T1 orthotopic tumor‐bearing mice were randomized into four groups (the blank control group with PBS, free DOX group, Fe‐MOF group, and DOX@Fe‐MOF group) when tumors reached ≈100 mm^3^. Treatment was carried out on days 0, 3, 6, and 9 at the dose of 5 mg kg^−1^. The metastasis process was monitored by bioluminescence imaging on an IVIS spectrum system after mice were intraperitoneally injected with d‐luciferin at fixed time points. Body weight and tumor volume were recorded every other day. At the end of treatment, the mice were humanely sacrificed and their main organs (kidney, lung, spleen, liver, heart, and tumor) were extracted for H&E and Masson stainings and TUNEL assay.

### Examination of Lung Metastasis

To examine lung metastasis, mice after various treatments were humanly sacrificed and their lungs were extracted and stained with India ink (15%). The lungs were then collected and soaked in Fekete's solution (100 mL 70% alcohol, 10 mL formalin, and 5 mL glacial acetic acid) at room temperature for 2‒3 d. The lungs were then measured under the microscope and metastatic tumors were divided into four grades (Grade I <0.5 mm, 0.5 mm ≤ Grade II < 1 mm, 1 mm ≤ Grade III < 2 mm, and Grade IV ≥ 2 mm), and then the number of metastases was normalized according to the following formula: I × 1 + II × 2 + III × 3 + IV × 4, where I, II, III, and IV represent the numbers of the four grades of metastases, respectively.

### Assessment of Systematic Toxicity

Healthy BALB/c mice were randomly divided into six groups and intravenously injected with 100 µL PBS (blank control), 100 µL 5 mg kg^−1^ DOX@Fe‐MOF, 100 µL 2.5 mg kg^−1^ free DOX, 100 µL 2.5 mg kg^−1^ Fe‐MOF, 100 µL 10 mg kg^−1^ Fe‐MOF, and 100 µL 20 mg kg^−1^ Fe‐MOF, respectively (*n* = 3). After one week, a standard biochemistry test was performed to evaluate liver/kidney function‐related indicators and blood panel parameters on a biochemical analyzer (iMagic‐M7) and a blood cell analyzer (BC‐31s, Mindray).

### Statistical Analysis

Statistical analysis was conducted using GraphPad Prism 8.3.0 software. Significance tests were performed using ordinary one‐way analysis of variance (ANOVA) with Dunnett's multiple comparisons test. *P < 0.05; **P < 0.01; ***P < 0.001; NS denotes no significant difference. All data are expressed as means ± standard deviation.

## Conflict of Interest

The authors declare no conflict of interest.

## Supporting information

Supporting Information

## Data Availability

The data that support the findings of this study are available from the corresponding author upon reasonable request.
