# Supplementary material for: Acid‐Degradable Hydrogen‐Generating Metal‐Organic Framework for Overcoming Cancer Resistance/Metastasis and Off‐Target Side Effects
Source: Adv Sci (Weinh). 2022 Jan 31;9(10):2101965. doi: 10.1002/advs.202101965 (PMC8981430; doi:10.1002/advs.202101965)
Supplement: Supplementary file 1 — Supporting Information [file ADVS-9-2101965-s001.pdf]

ADVANCED SCIENCE
Open Access

Supporting Information

for *Adv. Sci.*, DOI: 10.1002/advs.202101965

Acid-degradable hydrogen-generating metal-organic framework for overcoming cancer resistance/metastasis and off-target side effects

Xianxian Yao, Danyang Chen, Bin Zhao, Binru Yang, Zhaokui Jin, Mingjian Fan, Geru Tao, Shucun Qin, Wuli Yang, and Qianjun He**

Supporting Information

Acid-degradable hydrogen-generating metal-organic framework for overcoming cancer resistance/metastasis and off-target side effects

Xianxian Yao,[1,2] Danyang Chen,[2,3] Bin Zhao,[2] Binru Yang,[1] Zhaokui Jin,[2] Mingjian Fan,[2] Geru Tao,[4] Shucun Qin,[4] Wuli Yang,[1] and Qianjun He[2,3,4]**

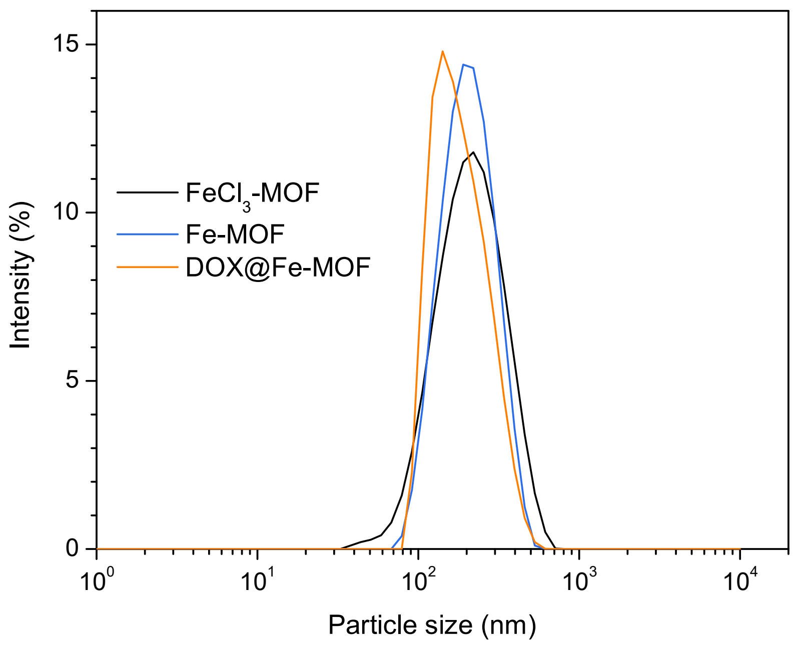

Figure S1. DLS size distribution of $FeCl_3$-MOF, Fe-MOF and DOX@Fe-MOF nanoparticles.

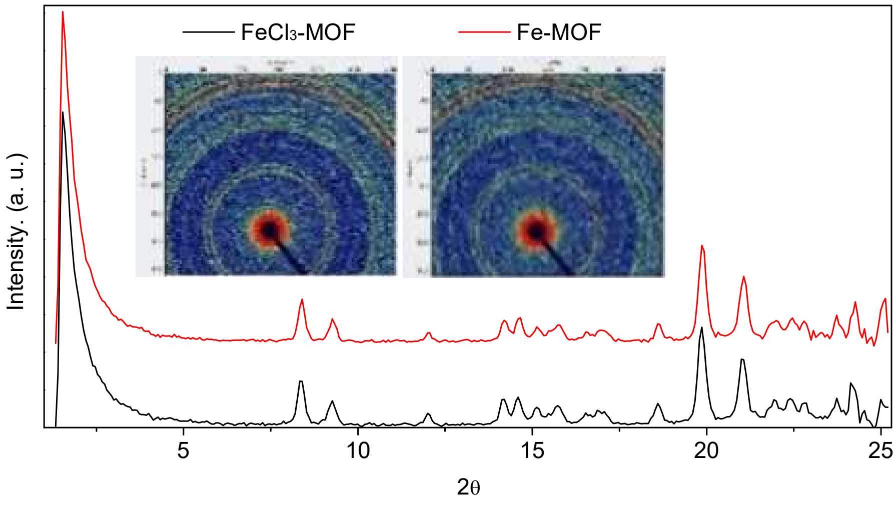

Figure S2. Small Angle X-ray Scattering (SAXS) of $FeCl_3$-MOF and Fe-MOF nanoparticles.

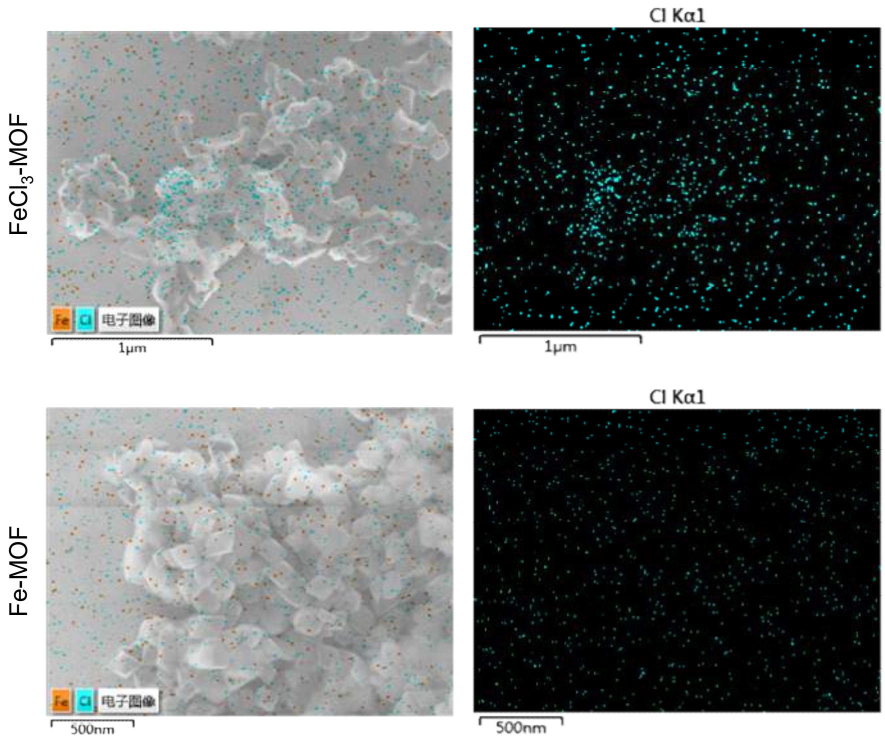

Figure S3. The elemental mapping images of $FeCl_3$-MOF and Fe-MOF to exhibit the removal of chlorine during the Fe-MOF synthesis.

Table S1. The lattice parameters of TPyP, $FeCl_3$-MOF and Fe-MOF nanocrystals calculated from powder XRD data in Figure 1e.

	**TPyP**	**$FeCl_3$-MOF**	**Fe-MOF**
crystal face	[301] [140]<br>[110] [020]	[301] [140]<br>[110] [020]	[301] [140]<br>[110] [020]
Θ_1	9.893	9.795	9.820
Θ_2	10.478	10.35	10.350
Θ_3	4.158	4.070	4.075
Θ_4	4.571	4.500	4.510
a	12.711 Å	13.018 Å	13.009 Å
b	19.324 Å	19.628 Å	19.585 Å
c	14.368 Å	14.482 Å	14.442 Å

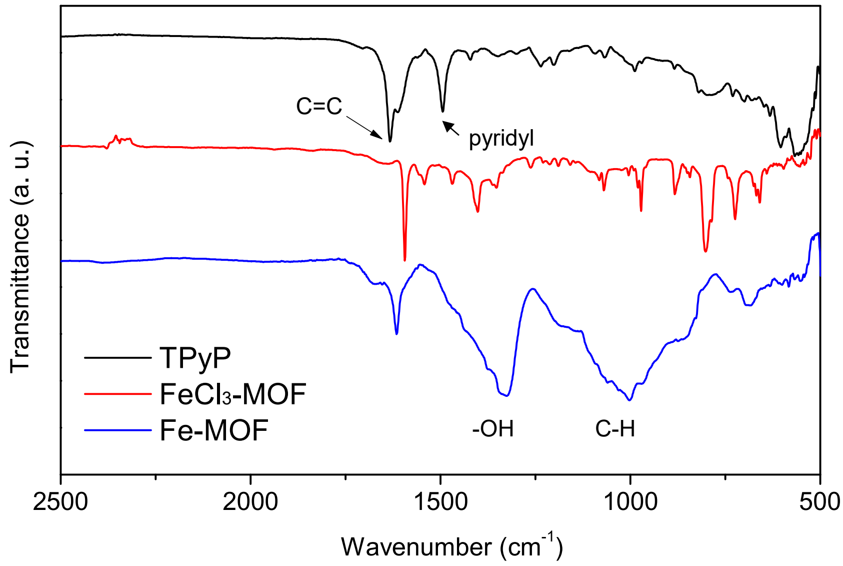

Figure S4. FT-IR spectra of TPyP, $FeCl_3$-MOF and Fe-MOF.

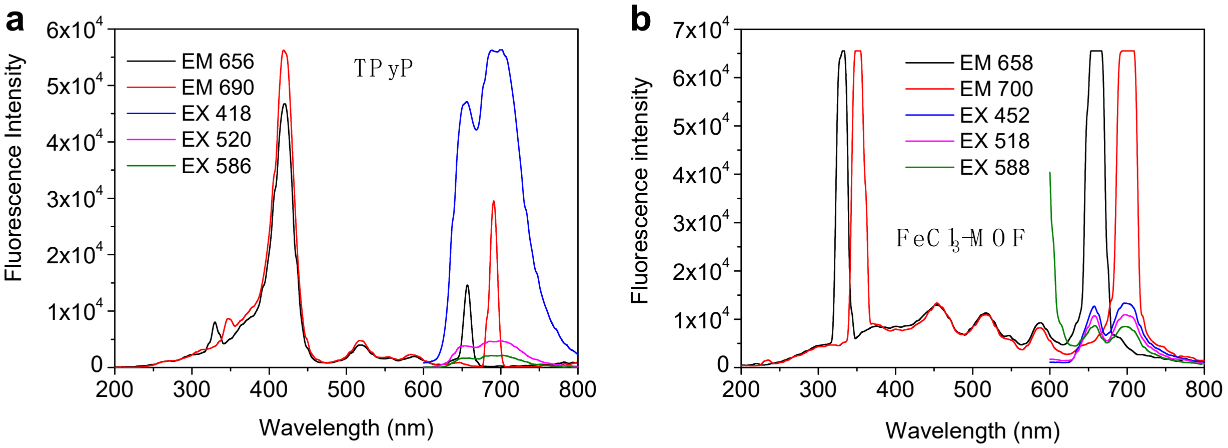

Figure S5. The fluorescence features of TPyP (**a**) and $FeCl_3$-MOF nanocrystals (**b**).

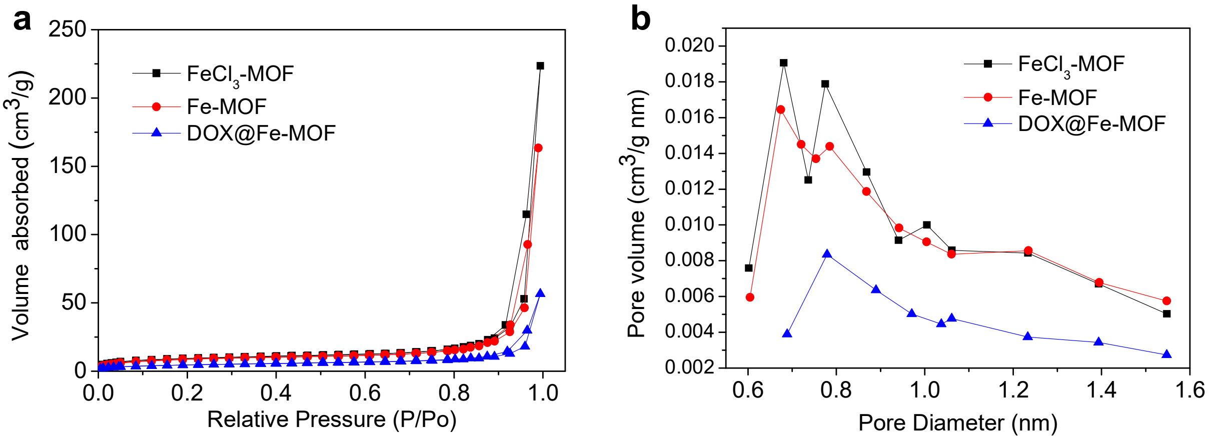

Figure S6. Nitrogen adsorption–desorption isotherms of $FeCl_3$-MOF, Fe-MOF and DOX@Fe-MOF (**a**), and the corresponding pore size distributions (**b**).

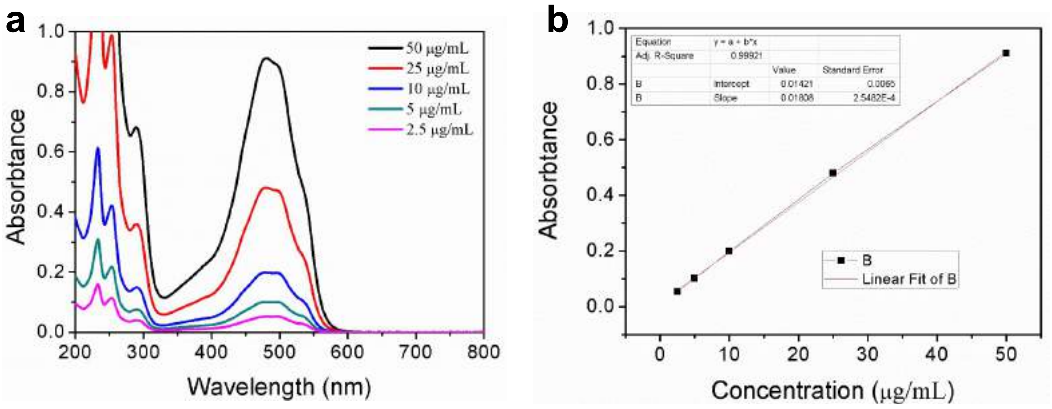

Figure S7. UV absorption spectra of DOX solutions at fixed different concentrations (**a**) and the corresponding calibration curve for calculation of DOX loading capacity of Fe-MOF nanocrystals.

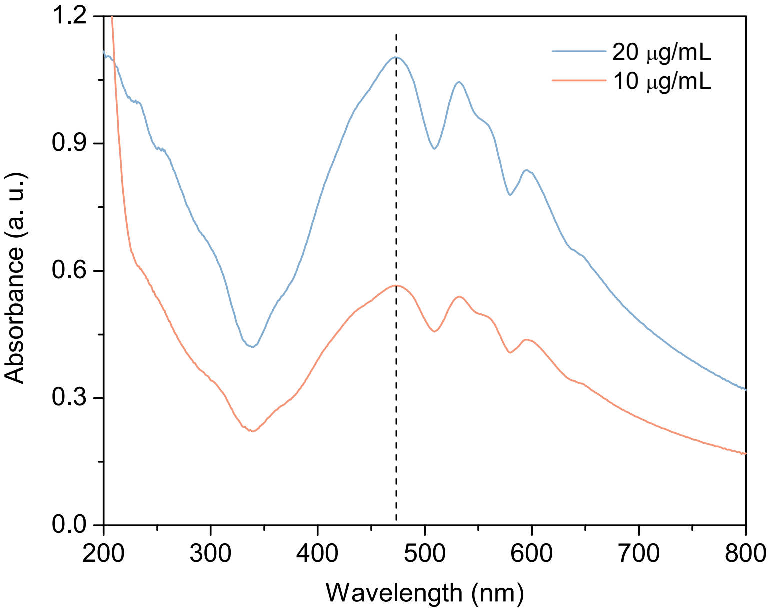

Figure S8. UV absorption spectra of DOX@Fe-MOF with concentrations of 10 μg/mL and 20 μg/mL.

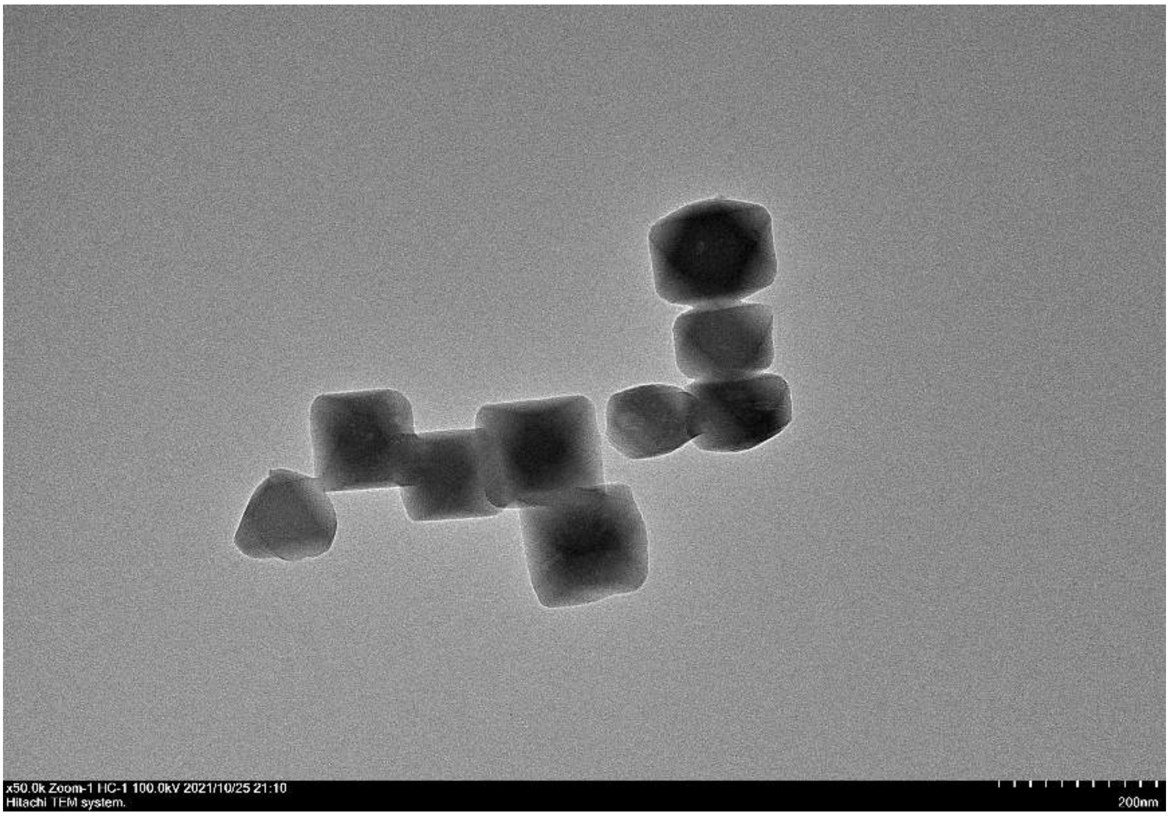

Figure S9. TEM image of DOX@Fe-MOF nanocrystals.

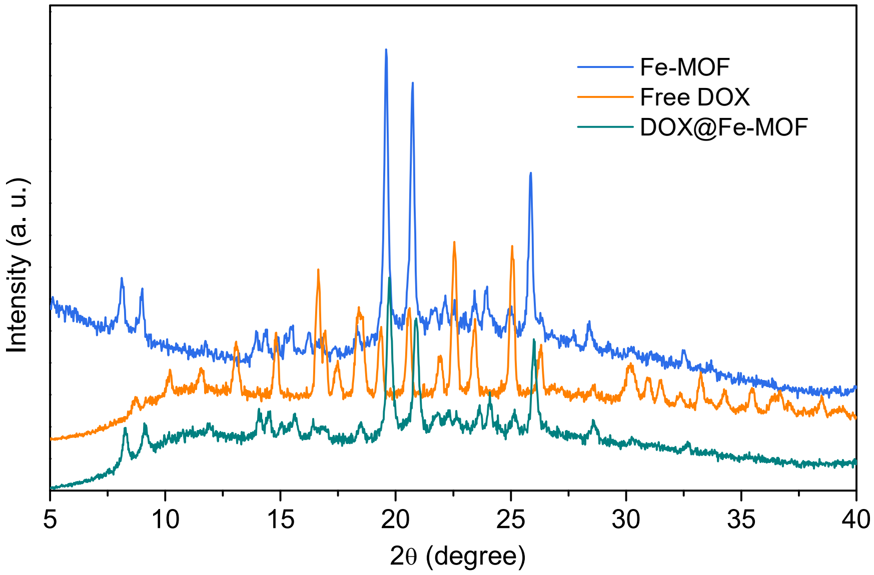

Figure S10. XRD patterns of DOX, Fe-MOF and DOX@Fe-MOF.

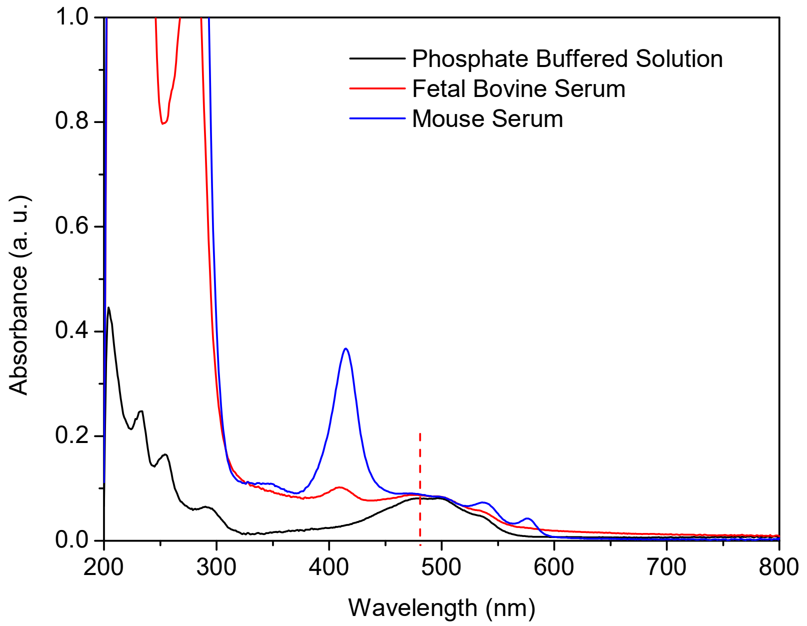

Figure S11. UV absorption spectra of DOX released from DOX@Fe-MOF in phosphate buffered solution, fetal bovine serum and mouse serum for 24 h.

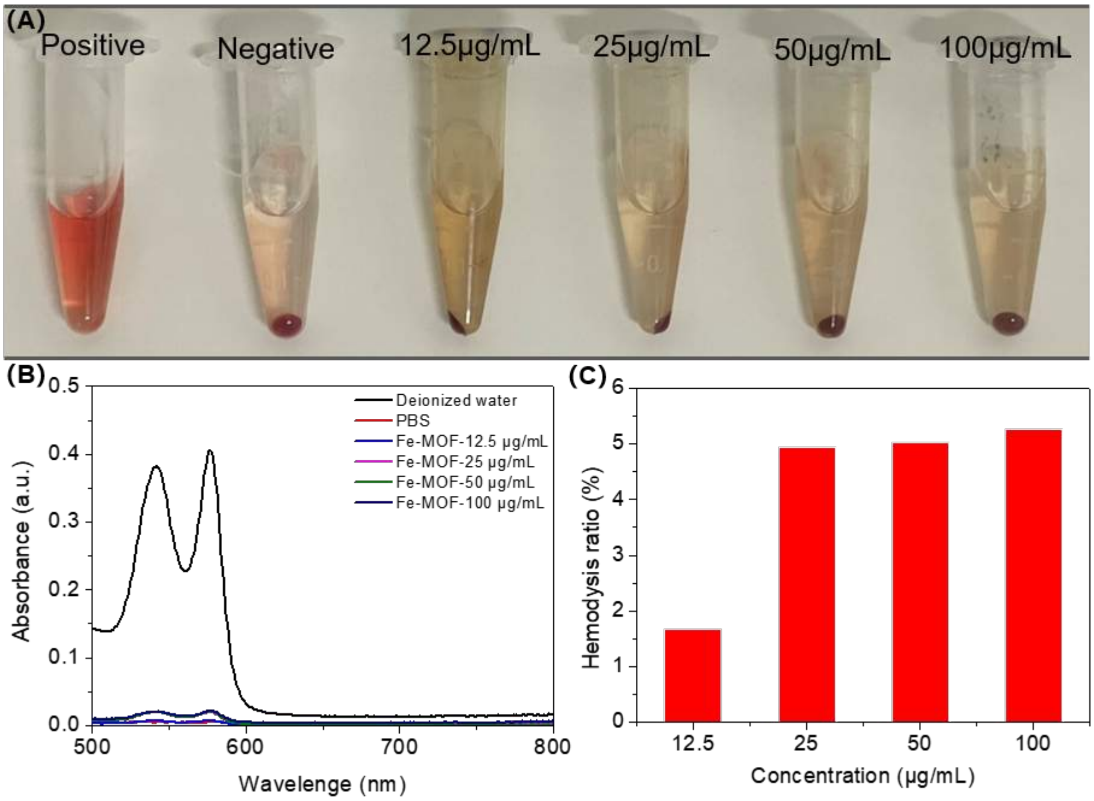

Figure S12. The hemolysis tests. Photographs (A), UV absorption spectra (B), and the percentage (C) of hemolysis of MRBCs in the presence of Fe-MOF at different concentrations ranging from 12.5 to 100 μg/mL for 3 h. Deionized water and PBS acted as positive and negative controls, respectively.

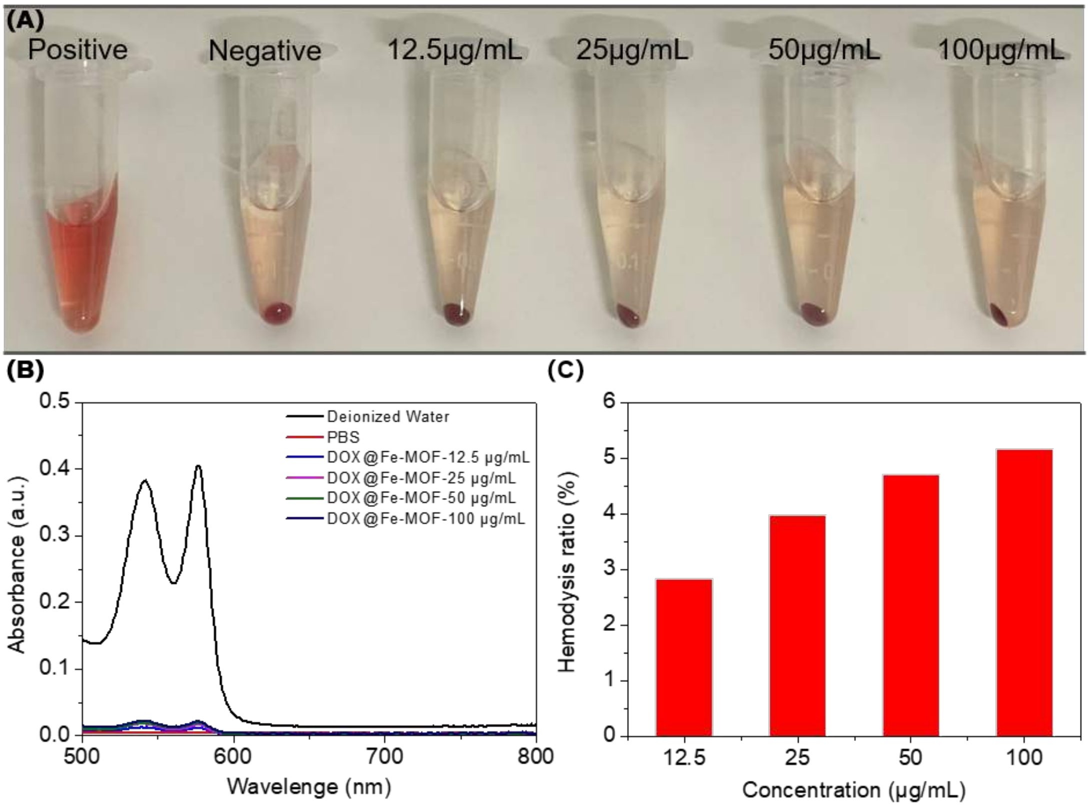

Figure S13. The hemolysis tests. Photographs (A), UV absorption spectra (B), the percentage (C) of hemolysis of MRBCs in the presence of DOX@Fe-MOF at different concentrations ranging from 12.5 to 100 μg/mL for 3 h. Deionized water and PBS acted as positive and negative controls, respectively.

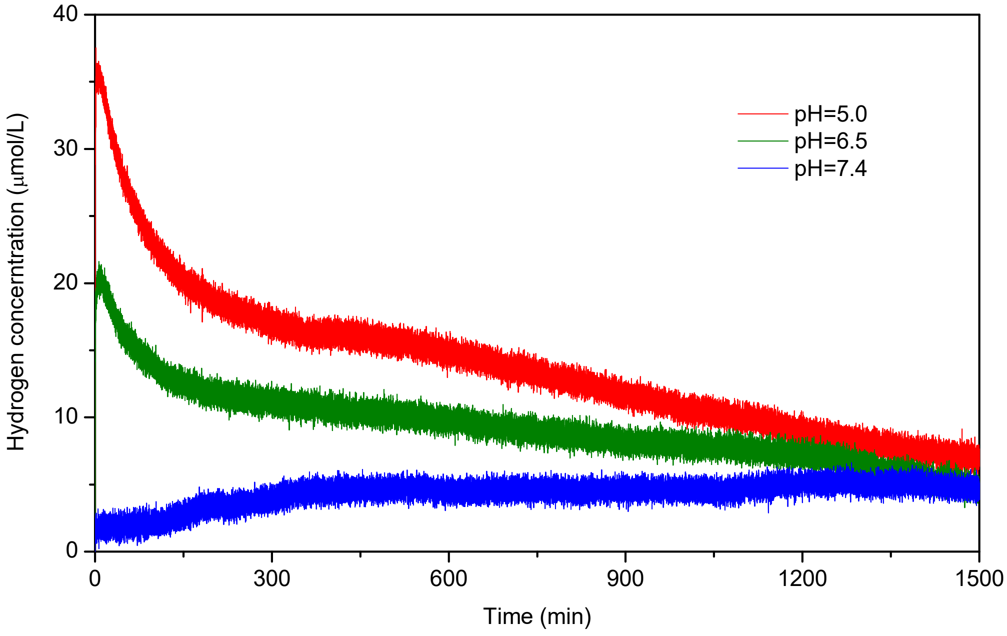

Figure S14. H_2 release behaviors of Fe-MOF nanocrystals at different pH environment.

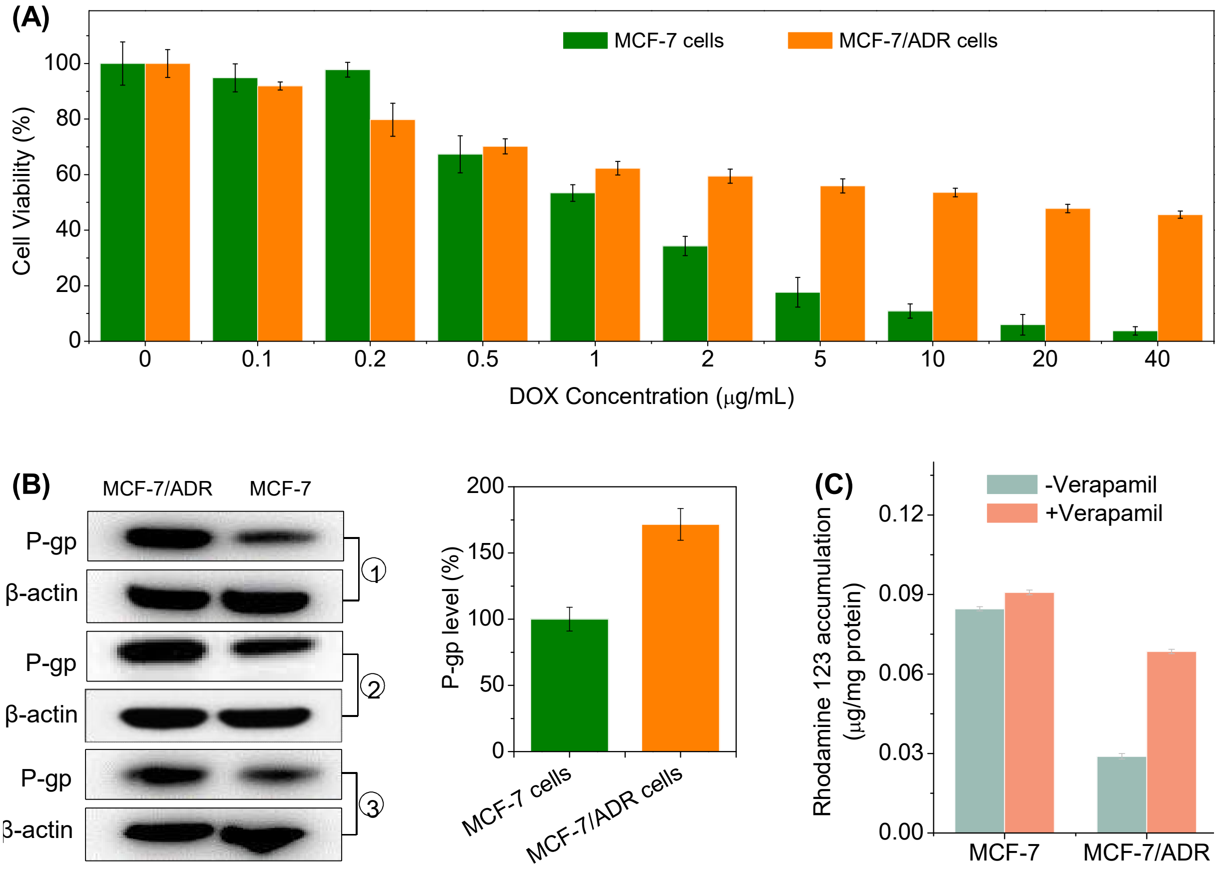

Figure S15. The viability of MCF-7 and MCF-7/ADR cells after 24 h of incubation with DOX (A), the intracellular P-gp level of MCF-7 and MCF-7/ADR cells (B) and the corresponding intensity of the P-gp expression (C) (1, 2, 3 represent three repeated experiments), and the intracellular accumulation of rhodamine 123 in MCF-7 and MCF-7/ADR cells (D).

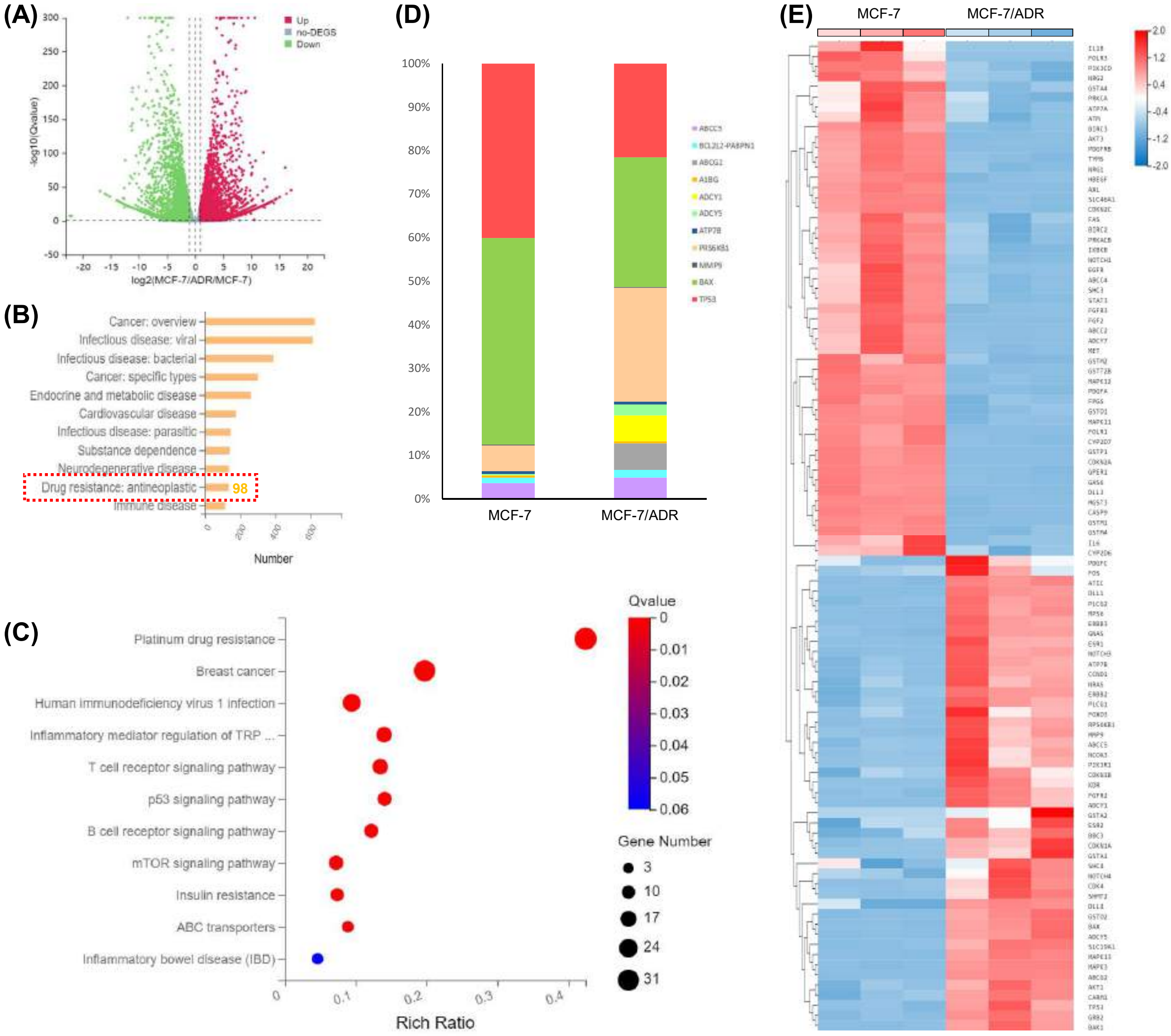

Figure S16. The volcano plot of gene expression between MCF-7 and MCF-7/ADR cells (A), KEGG pathway (human diseases) enrichment analysis (B) and GO enrichment analysis (C) of differentially expressed genes between MCF-7 and MCF-7/ADR cells, the relative change of differentially expressed gene expression between MCF-7 and MCF-7/ADR cells (D), the heat map of 98 differentialy expressed genes related to drug resistance between MCF-7 and MCF-7/ADR cells (E).

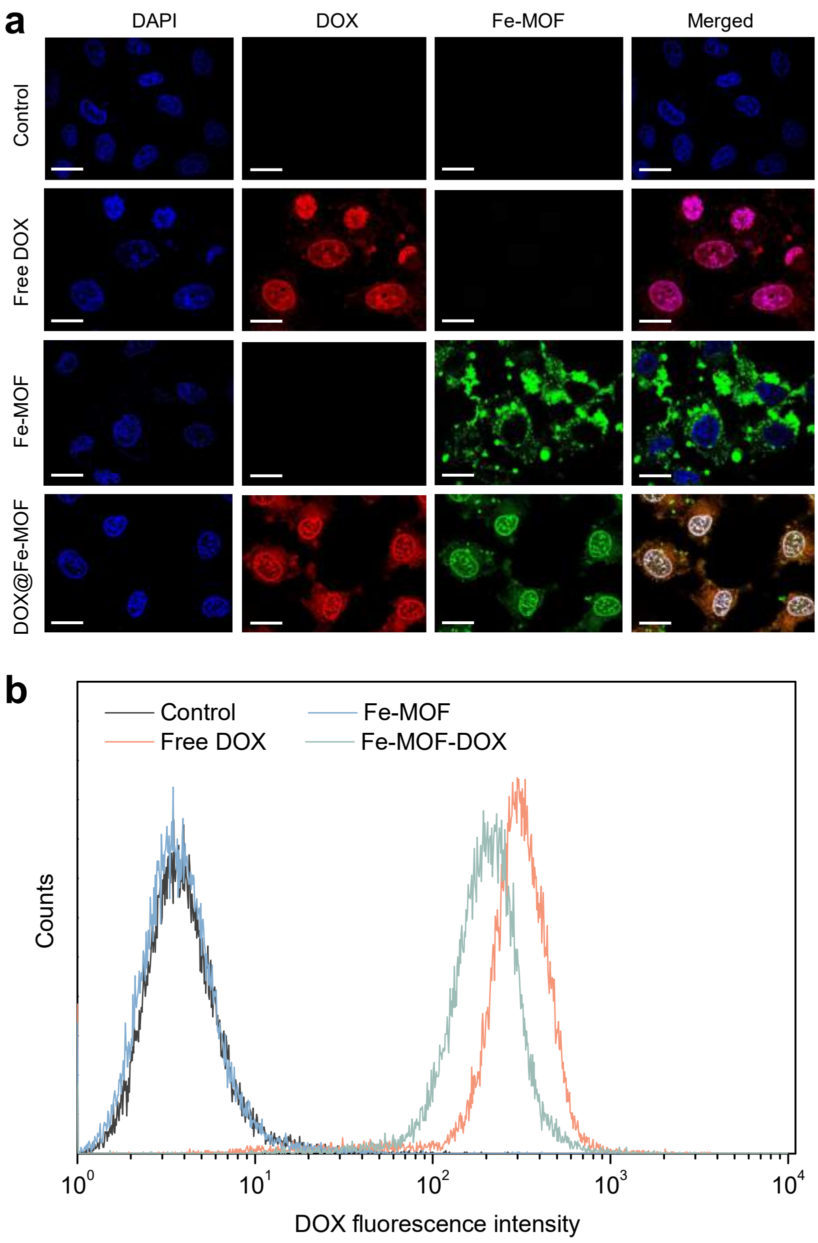

Figure S17. CLSM (**a**) and flow cytometry analyses (**b**) of nanoparticles after incubation for 4 h by MCF-7 cells. The scale bar corresponds to 30 μm.

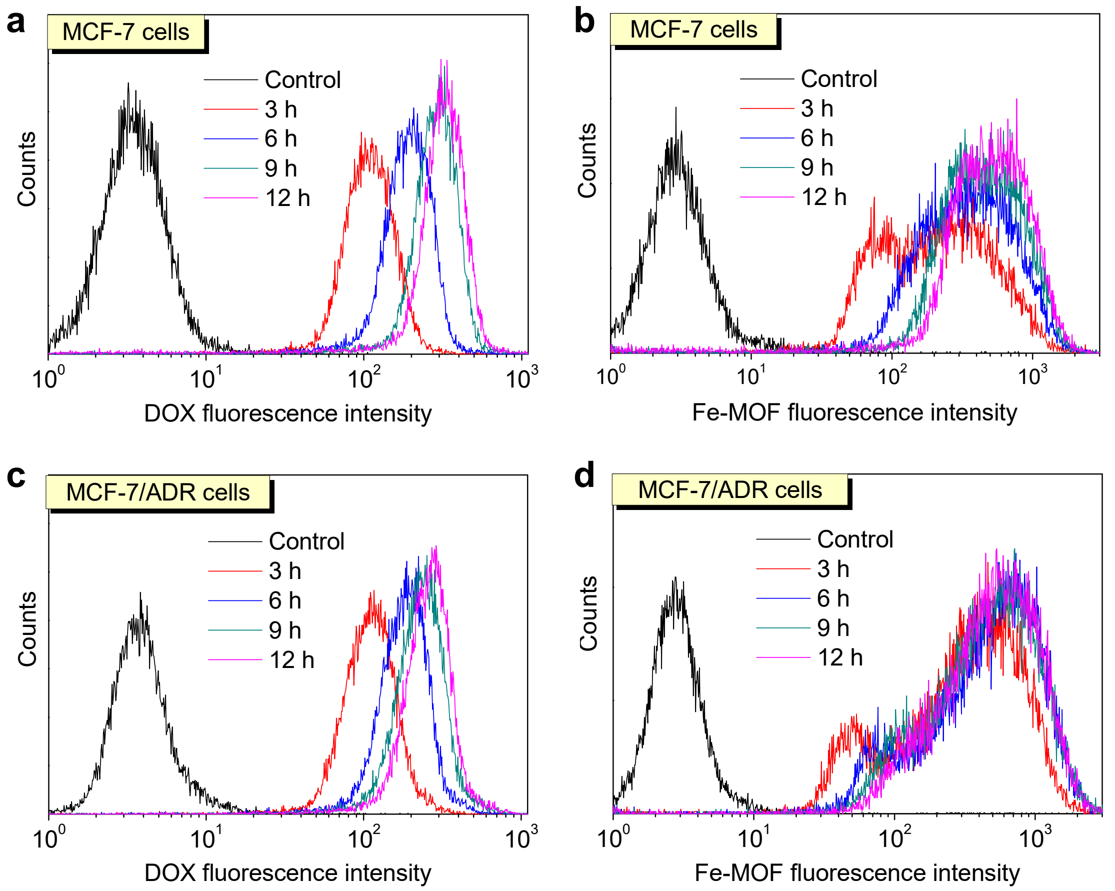

Figure S18. Flow cytometry analyses of the DOX@Fe-MOF nanomedicine after incubation different time by MCF-7 and MCF-7/ADR cells.

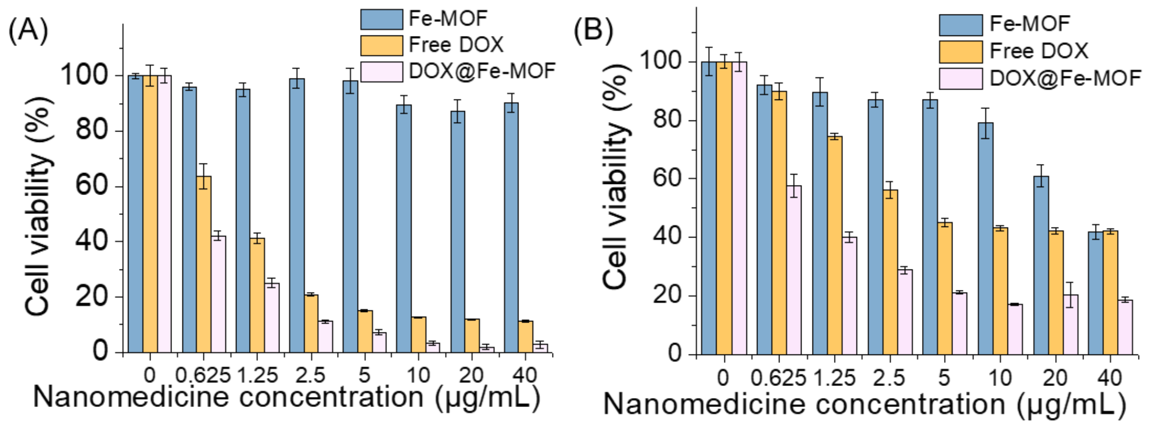

Figure S19. The cytotoxicity of nanocrystals against MCF-7 cells (A) and MCF-7/ADR cells (B) after incubation for 48 h.

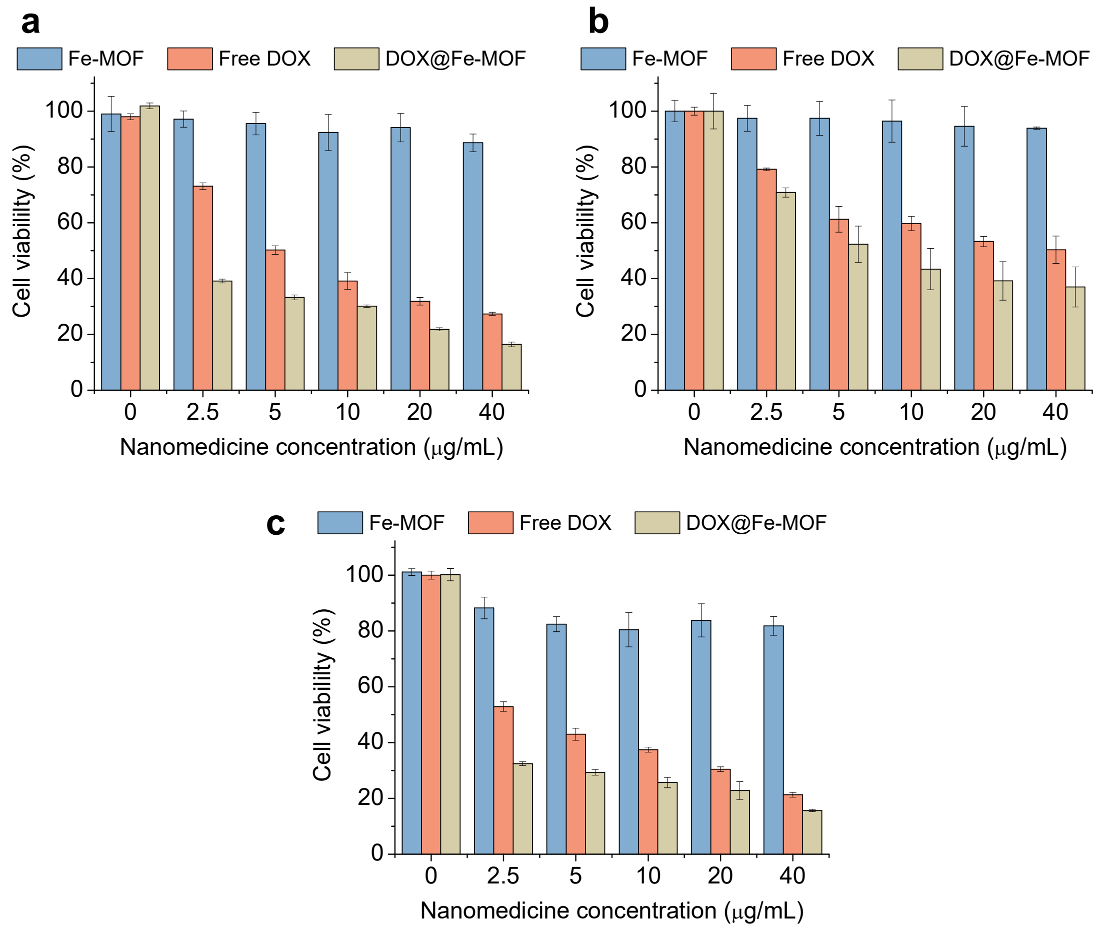

Figure S20. The cytotoxicity of free MOF, Fe-MOF and DOX@Fe-MOF against HeLa (**a**), A549 (**b**) and 4T1 cells (**c**).

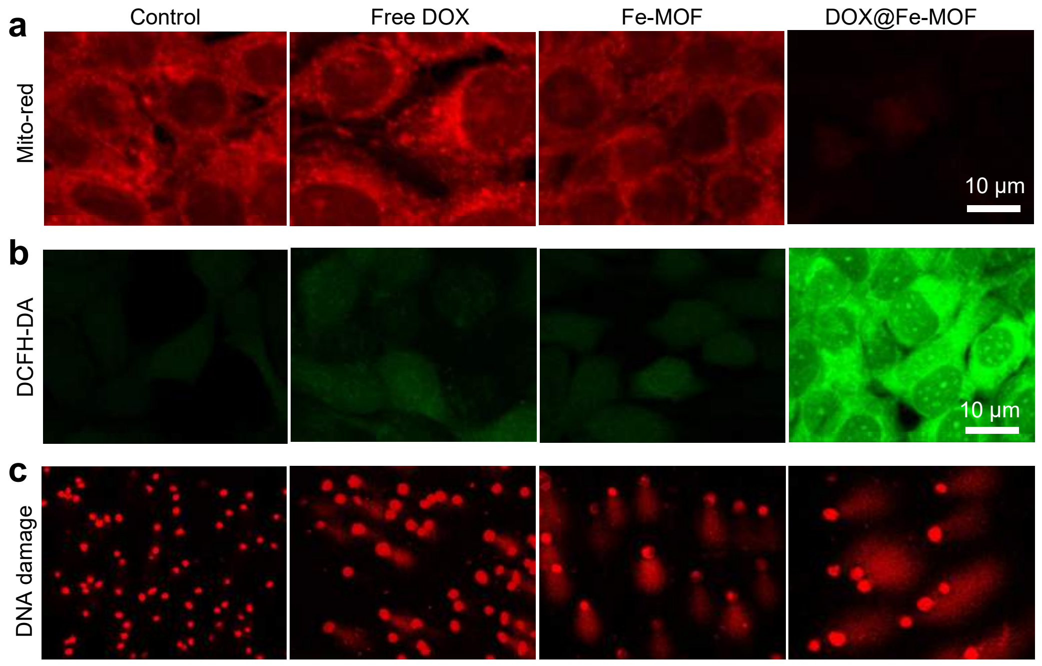

Figure S21. CLSM of mitochondrial membrane potentials (**a**), reactive oxygen species (ROS) generation (**b**), and DNA damage (**c**) of 4T1 cells.

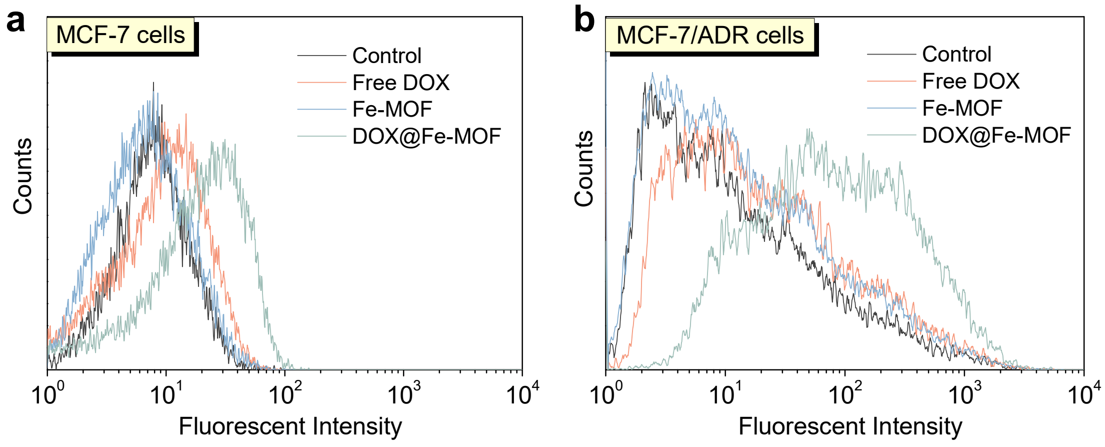

Figure S22. Flow cytometry analyses of reactive oxygen species generation in MCF-7 (**a**) and MCF-7/ADR cells (**b**).

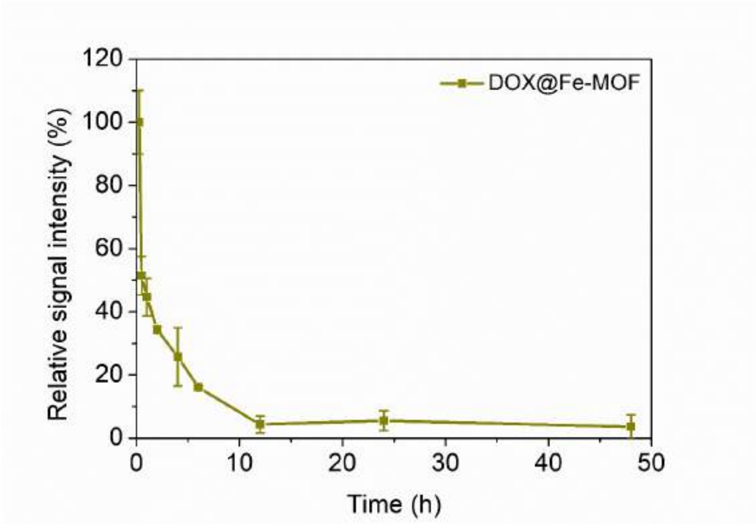

Figure S23. *In vivo* blood retention profile of DOX@Fe-MOF (1 mg/mL, 100 µL).

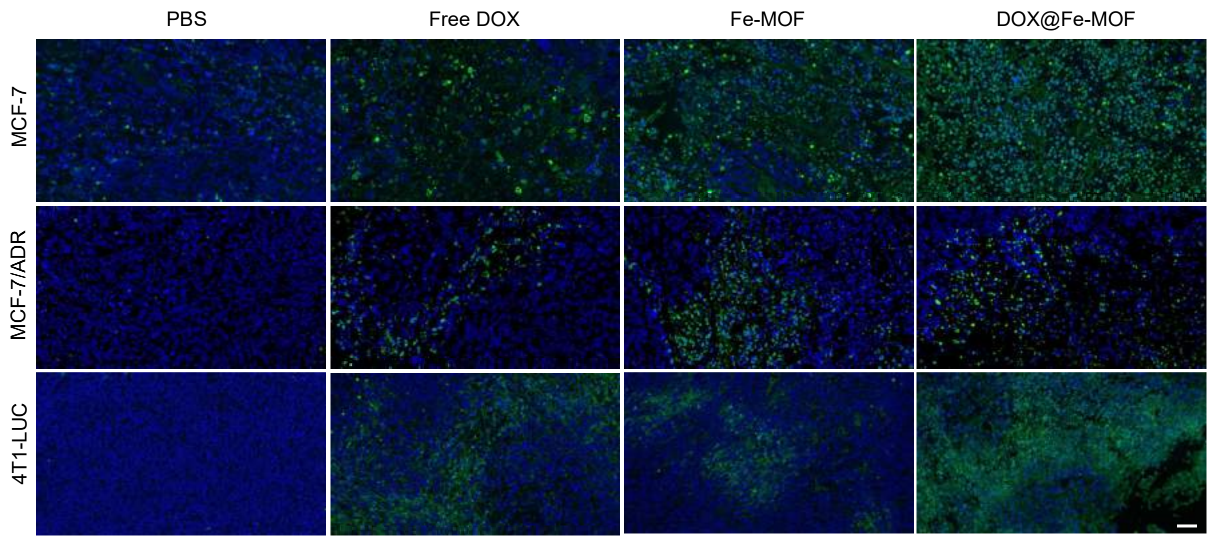

Figure S24. TUNEL staining of tumor tissues. The scale bar corresponds to 50 µm.

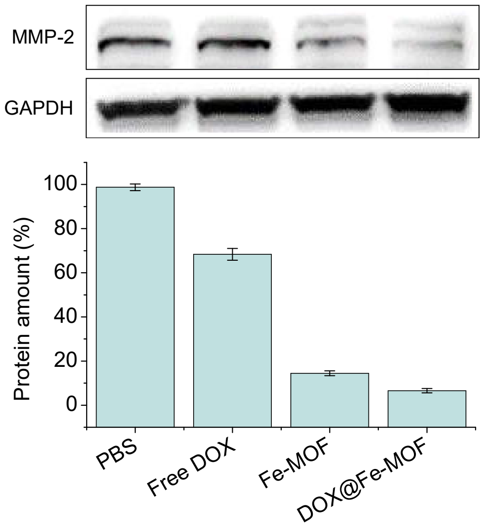

Figure S25. The levels of MMP-2 in 4T1 cells after various treatments detected by Western blot where GAPDH was a loading control.

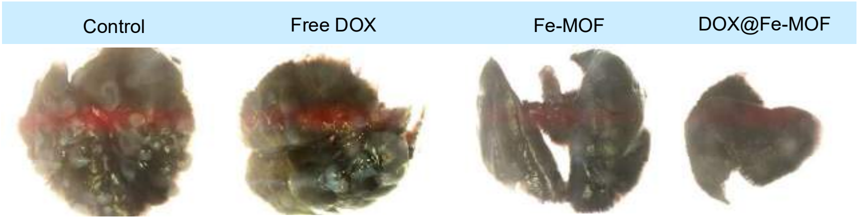

Figure S26. Representative lung metastasis nodules in different groups at the end of treatment.

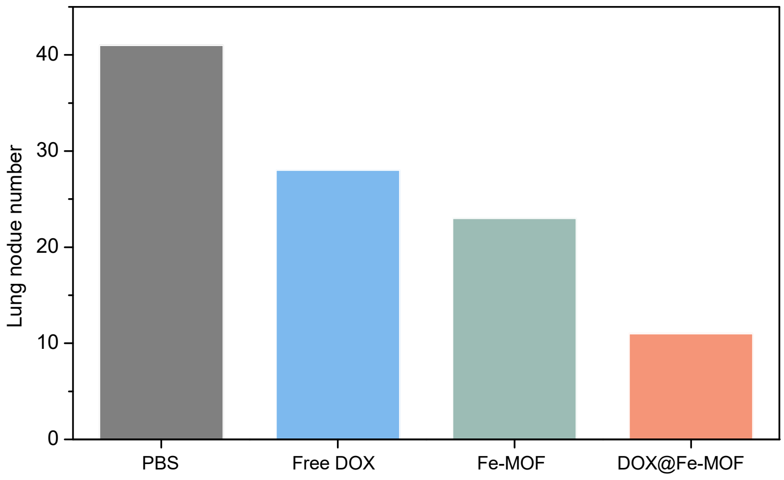

Figure S27. The number of lung metastasis nodules in different groups at the end of treatment.

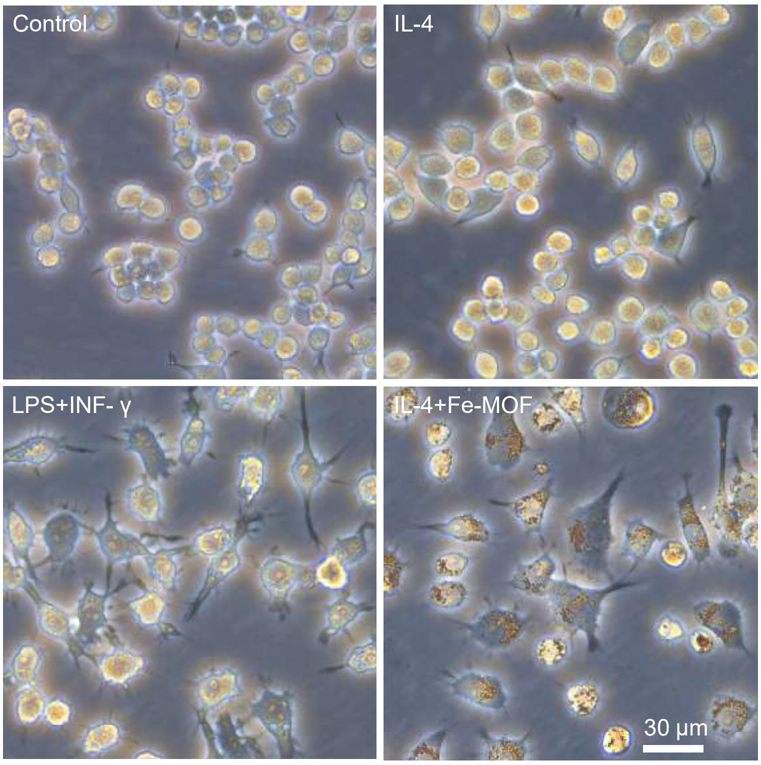

Figure S28. The morphology of raw264.7 macrophages after individually treated with PBS (control), IL-4 (positive control for M2-phenotype), LSP+INF-γ (positive control for M1-phenotype), and and IL-4 plus Fe-MOF (M2-to-M1 repolarization). The scale bar corresponds to 30 μm.

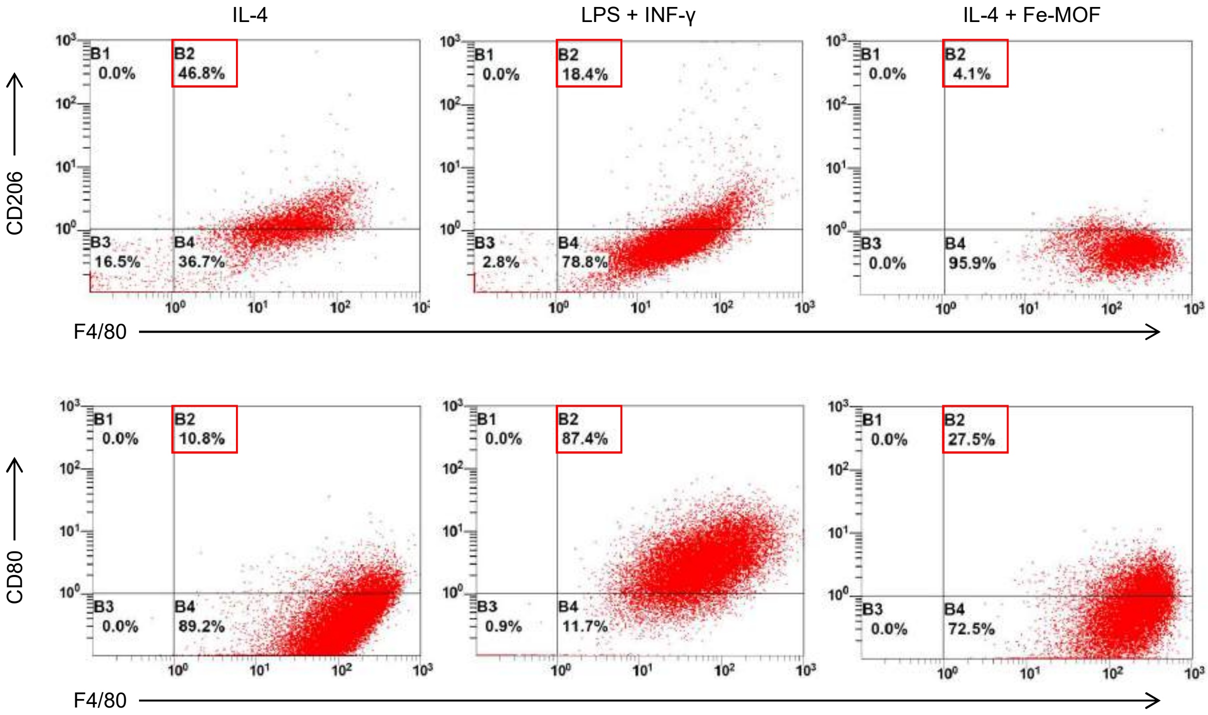

Figure S29. Flow cytometric analysis of M2-phenotype (CD206) and M1-phenotype (CD80) contents in raw264.7 macrophages after individually treated with PBS (control), IL-4 (positive control for M2-phenotype), LSP+INF-γ (positive control for M1-phenotype), and IL-4 plus Fe-MOF (M2-to-M1 repolarization).

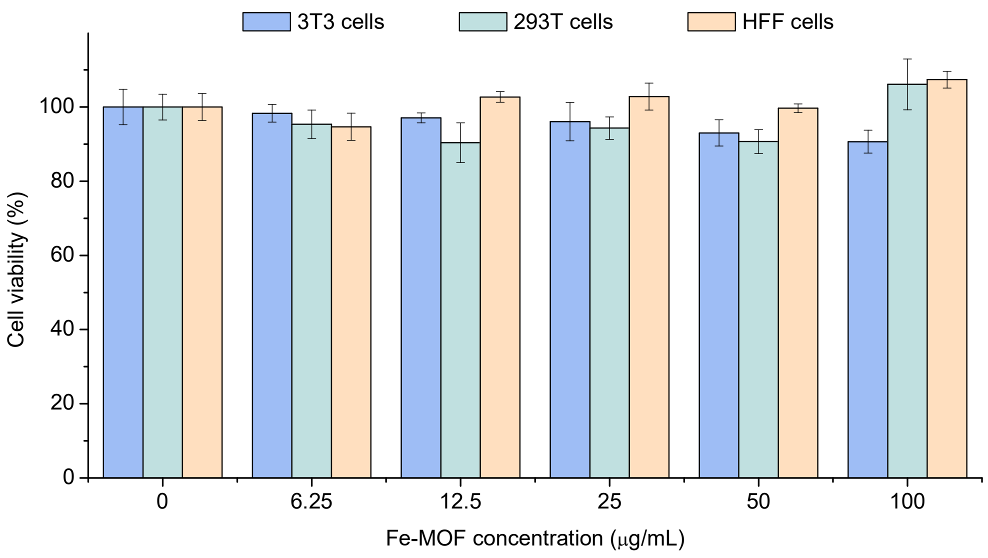

Figure S30. The cytotoxicity of Fe-MOF nanoparticles to normal cells including 3T3, 293T and HFF cells.

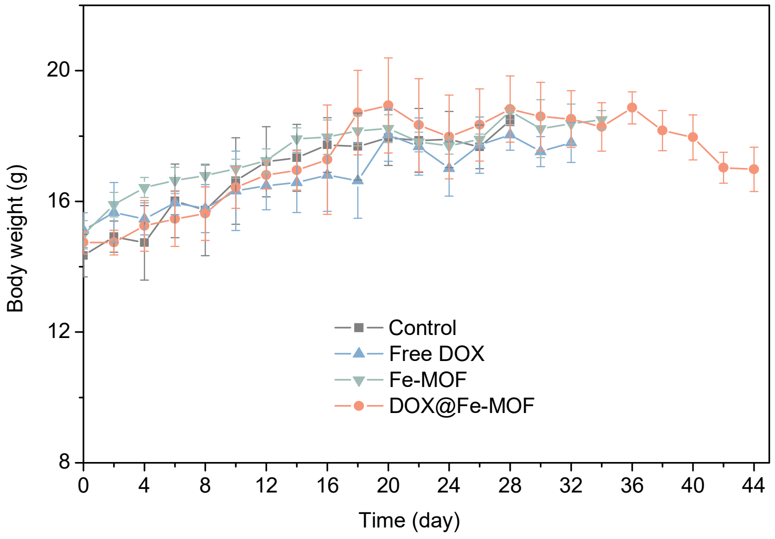

Figure S31. The body weight comparison after lung metastasis tumors treatment.

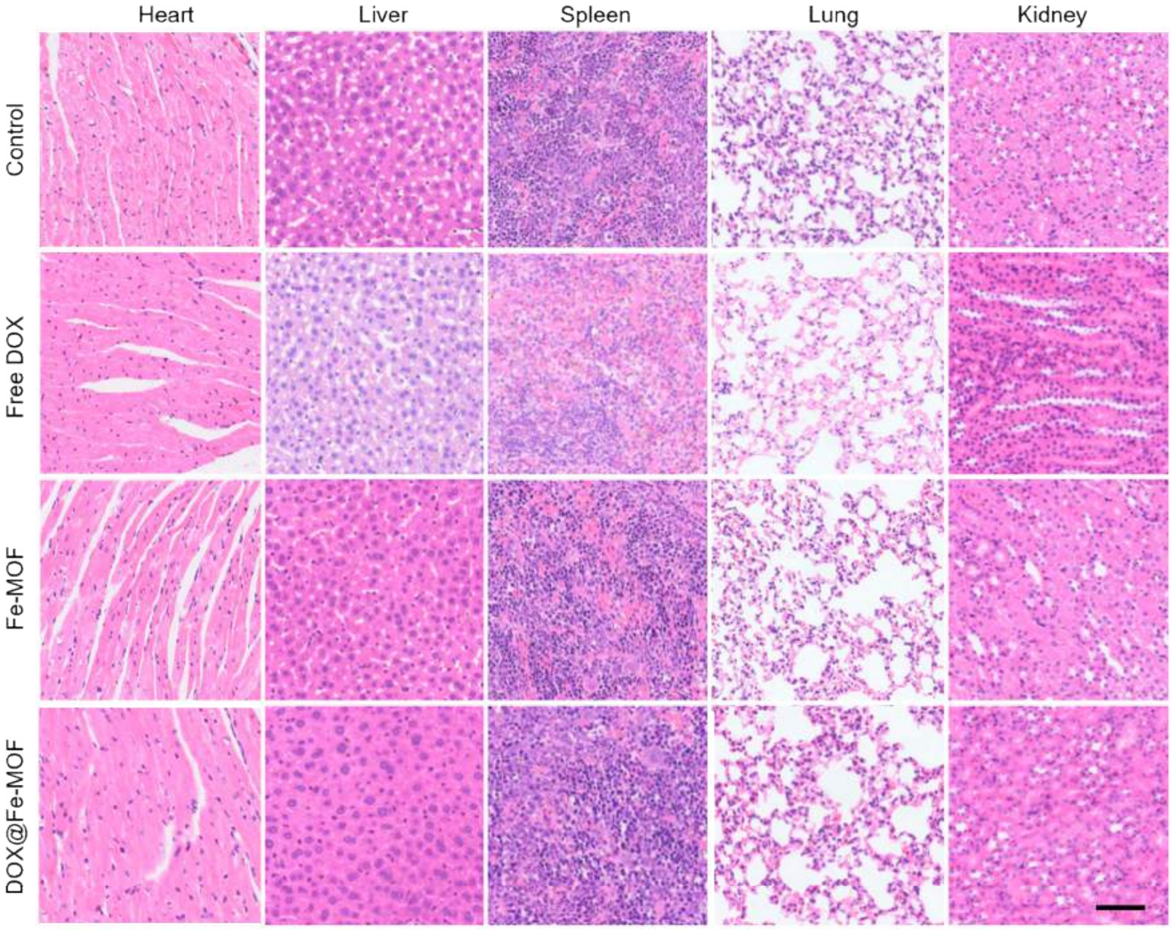

Figure S32. The histological analysis of main organs of treated MCF-7 tumor-bearing mice by the H&E staining method. The scale bar corresponds to 200 μm.

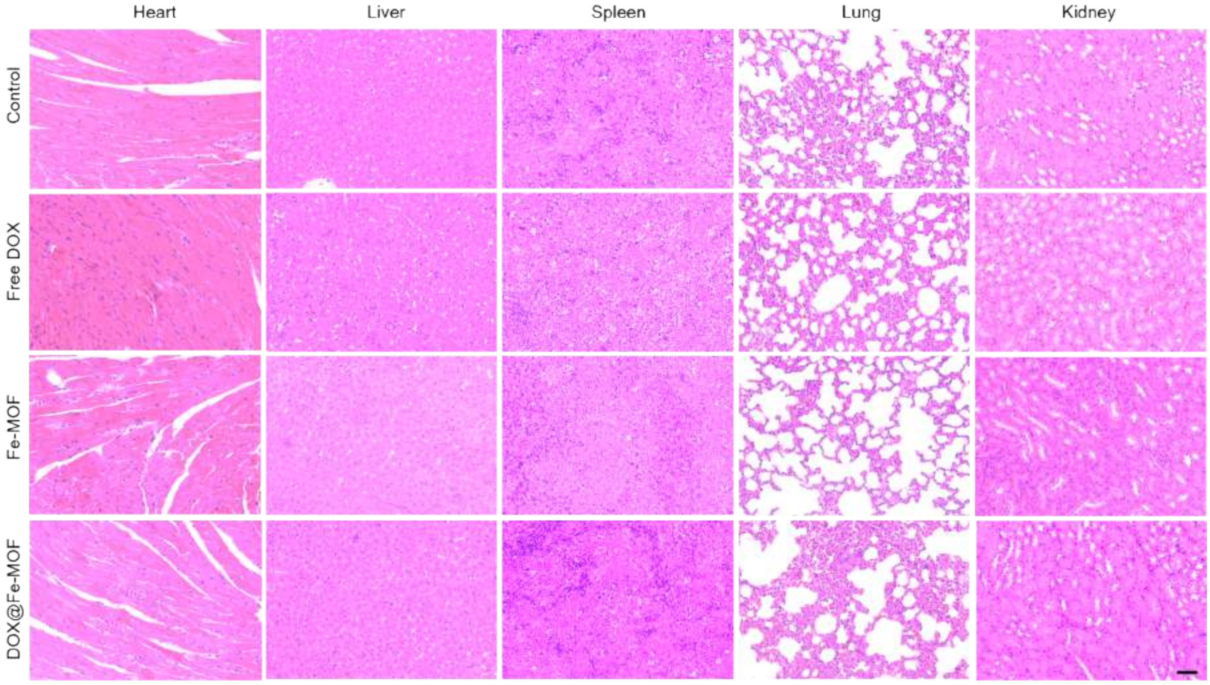

Figure S33. The histological analysis of main organs of treated MCF-7/ADR tumor-bearing mice by the H&E staining method. The scale bar corresponds to 50 μm.

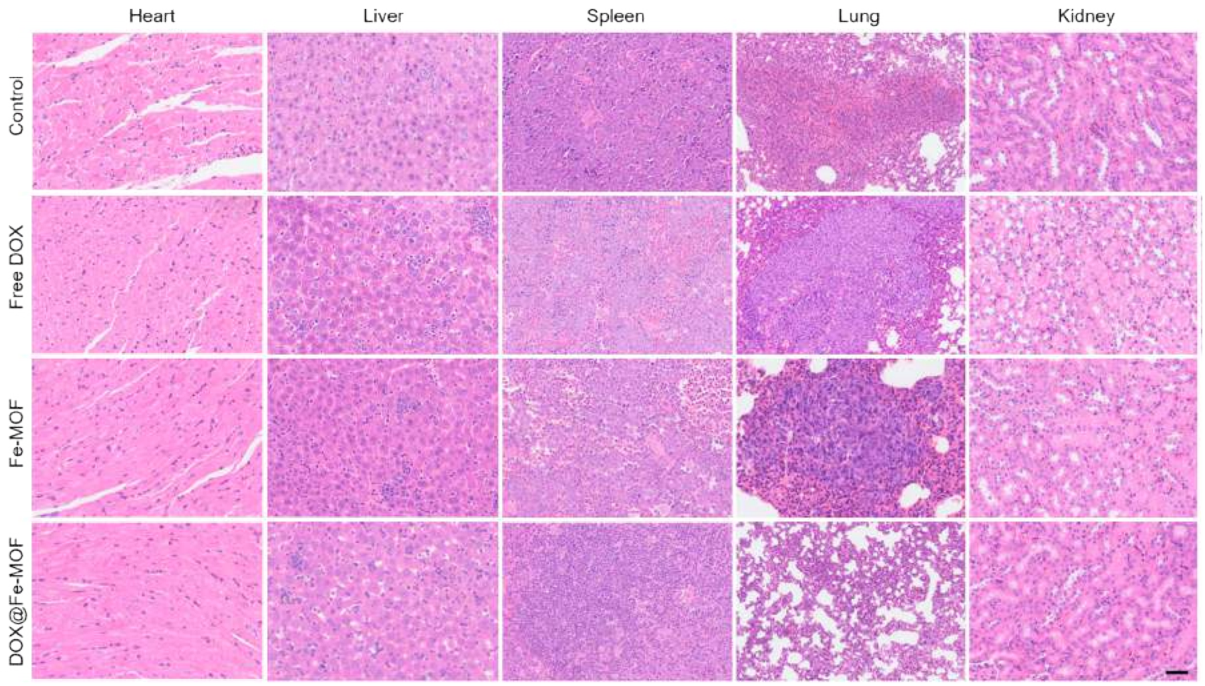

Figure S34. The histological analysis of main organs of treated 4T1 tumor-bearing mice by the H&E staining method. The scale bar corresponds to 50 μm.

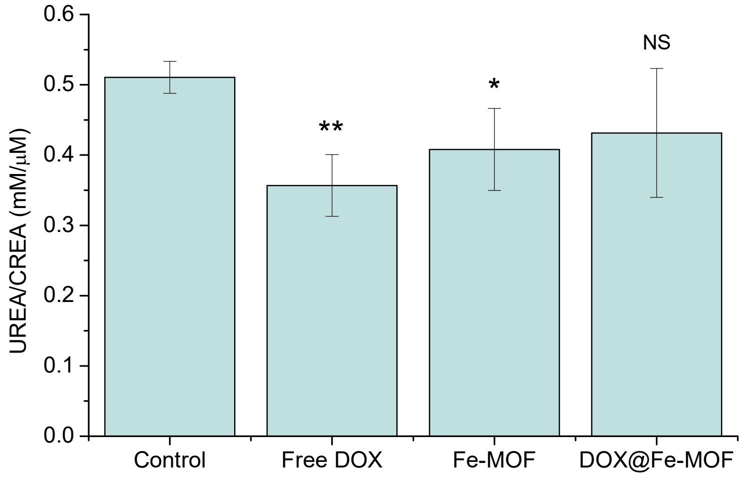

Figure S35. The ratio of UREA/CREA in treated 4T1 mice for one week. *P* values were calculated by the two-tailed Student's *t*-test (**P=0.005712, *P=0.047377; NS, no significant difference).
